# Abietane-Type Diterpenoids From *Nepeta bracteata* Benth. and Their Anti-Inflammatory Activity

**DOI:** 10.3389/fchem.2022.944972

**Published:** 2022-07-04

**Authors:** Er-Lan Yang, Yong Hou, Guo-Xu Ma, Lin-Jun Zou, Xu-Dong Xu, Hai-Feng Wu, Jun-Shan Yang, Hong-Wan Wei, Cong-Zhao Fan, Zhao-Cui Sun, Lei-Ling Shi

**Affiliations:** ^1^ Institute of Medicinal Plant Development, Chinese Academy of Medical Sciences and Peking Union Medical College, Peking, China; ^2^ Xinjiang Institute of Chinese and Ethnic Medicine, Urumqi, China

**Keywords:** abietane-type diterpenoids, ursane-type triterpenoid, *Nepeta bracteata* Benth., anti-inflammatory, drug candidates

## Abstract

Terpenes possess a wide range of structural features and pharmaceutical activities and are promising for drug candidates. With the aim to find bioactive terpene molecules, eight new compounds were isolated from the medicinal plant *Nepeta bracteata* Benth., including seven new abietane-type diterpenoids **(1–7)**, along with a new ursane-type triterpenoid **(8)**. The structures of compounds **1–8** were elucidated through the detailed spectroscopic analyses of their 1D and 2D NMR and MS data, and the absolute configurations of compounds **1–7** were determined by comparing their experimental and calculated ECD spectra. Compound **1** was a novel degraded carbon diterpene with the disappearing of methyl signal at C-19, while compound **7** possessed a new norabietane-type diterpenoid carbon skeleton with the presence of five-membered lactone arising from ring rearrangement. The anti-inflammatory of all obtained isolates were evaluated on lipopolysaccharide (LPS)-stimulated RAW 264.7 cells and the results of anti-inflammatory activity screening showed that compared with the LPS model group, all compounds were significantly down-regulation the TNF-α inflammatory factor at the specific concentration, except for compound **6**.

## Introduction

Inflammation is a complex set of interactions among soluble factors and cells that can arise in any tissue in response to traumatic, infectious, post-ischaemic, toxic or autoimmune injury, and is closely related to many diseases such as arthritis, psychosis, cardiovascular and cerebrovascular diseases, cancer, obesity-linked inflammatory diseases diabetes, fatty liver disease, airway inflammation, and atherosclerosis (Nathan, 2002; Wellen and Hotamisligil, 2005). Although non-steroidal anti-inflammatory drugs (NSAIDs) play an important role in the clinical treatment of anti-inflammatory, such as aspirin, ibuprofen and naproxen, they may thus cause a wide range of adverse effects, such as damaging to the gastrointestinal tract and leading to renal, cardiovascular and liver dysfunction (Bacchi et al., 2012). Therefore, it is extremely important to find effective anti-inflammatory drugs with fewer side effects. Natural products are important sources of inspiration and key resources for drug discovery. Finding leading compounds from medicinal plants is not only a traditional way, but also an effective and convenient method.

*Nepeta bracteata* Benth., a dry whole grass of *N. bracteata* Benth. in the family Lamiaceae, the Uyghur name is “Zofa”, which is an imported medicinal material commonly used in Xinjiang Uyghur Hospital and mainly distributed in Pakistan, Iran, Nepal and other countries (Latif et al., 2013; Siddiqui et al., 2017). *N. bracteata* Benth. possesses various pharmacological effects, including generating heat, warming the lungs and relieving asthma, dispeling cold and relieving cough, dring dampness and removing phlegm, sweats and detoxifies, reducing inflammation and swelling, and mainly treats damp-cold and mucous respiratory diseases (Naghibi et al., 2005; Quddus et al., 2009; Sehar et al., 2015). While modern pharmacology has shown that its extract has significant anti-inflammatory activity (Wang et al., 2016), there are few related researches have been conducted to characterize its chemical components. In our continuous effort to discover novel bioactive constituens from *N. bracteata* Benth., four new abietane-type diterpenoids (nepetabrates A-D), one amide alkaloid, and five known diterpenoids were isolated (Zhang et al., 2021). Abietane-type diterpenoids are a structural active class of phytometabolites with largely nonpolar structures and are the structural basis of a variety of natural compounds, such as rosin acid, carnosic acid, etc (Brückner et al., 2014; González, 2015). Meanwhile, they possess a highly diverse repertoire of bioactivities, such as anti-ulcer, anti-tumor, anti-malarial, antibacterial, antimicrobial, antineuro inflammatory, antiviral activity, etc (González, 2015). Considering its remarkable activities, we further investigated the abietane-type diterpenoids in *N. bracteata* Benth.

**FIGURE 1:**
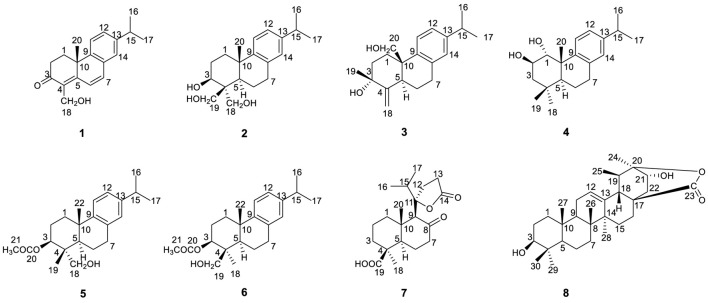
Chemical structures of compounds **1–8**.

In the present study, eight new compounds were isolated and characterized using spectroscopic and chromatographic methods, including six new abietane-type diterpenoids, nepetabrates E-J (**1–6**), a novel norabietane-type skeleton with five-membered lactone, nepetabrate K (**7**) as well as a new ursane-type triterpenoid, vanguerolactone (**8**) (Figure 1). The absolute configurations of compounds **1**-**7** were determined by comparing their experimental and calculated ECD spectra. Herein, the isolation and structural elucidation of new isolates from *N. bracteata* Benth. as well as their anti-inflammatory activities are reported.

## Experimental Section

### General Experimental Procedures

Optical rotations were measured with an Anton Paar MCP200 polarimeter (Anton PaarGmbH, Graz, Austria). UV (1.0 mg of sample was dissolved in 3 ml of chromatographic grade methanol for each sample) and IR (1.0 mg of sample was pressed in KBr for each sample) spectral data were recorded on Shimadzu UV2550 and FTIR-8400S spectrometers (Shimadzu, Kyoto, Japan). ECD spectra were recorded using an Applied Photophysics Chirascan spectro polarimeter (Chirascan, Surrey, United Kingdom). 1D and 2D NMR spectra were obtained using a Bruker A V III 600 NMR spectrometer with chemical shift values presented as δ values using TMS as the internal standard (samples dissolved in an appropriate amount of deuterated chloroform). HR-ESI-MS was performed using an LTQ-Orbitrap XL spectrometer (Thermo Fisher Scientific, Boston, MA, United States); samples were dissolved in chromatographic methanol and treated through a membrane, single pump. Column chromatography (CC) was performed using silica gel (100–200 and 200–300 mesh, Qingdao Marine Chemical Plant, Qingdao, China). Semi-preparative HPLC was performed using an HPLC PUMP K-501, LC3000 high-performance liquid chromatograph (Beijing Tong Heng Innovation Technology Co., Ltd, Beijing, China), and Kromasil 100-5C18, 250 × 10 mm, E108850. Precoated silica gel GF254 plates (Zhi FuHuang Wu Pilot Plant of Silica Gel Development, Yantai, China) were used for TLC. All solvents used (petroleum ether, ethyl acetate, dichloromethane, methanol (analytical grade and chromatographic grade), and deuterated chloroform) were of analytical grade (Beijing Chemical Plant, Beijing, China).

### Plant Material

*Nepeta bracteata* Benth. was purchased from Xinjiang Uygur hospital (Urumqi, China) and identifified as *Nepeta bracteata* Benth. by Professor Leiling Shi. The certified medicinal material specimen (M20191025) was kept in the medicinal material room of Xinjiang Institute of Chinese and Ethnic Medicine (Urumqi, China).

### Extraction and Isolation

The aerial part of *Nepeta bracteata* Benth. (6.0 kg) was soaked in ethanol at room temperature (3 × 40 L, 3 h each time) and extracted three times under reflux. Removal of the ethanol under reduced pressure yielded the ethanol extract (437.0 g). The ethanol extract was dissolved in water and successively extracted with petroleum ether (PE, 3 × 1000 ml), dichloromethane (CH_2_Cl_2_, 3 × 1000 ml) and ethyl acetate (EtOAc, 3 × 1000 ml). The petroleum fraction (134.8 g) was subjected to column chromatography (CC) over a silica gel (100–200 mesh), eluting with a step wise gradient of PE/EtOAc (from 1:0 to 0:1; that is, 1:0, 100:1, 50:1, 25:1, 8:1, 5:1, 1:1, and 0:1, v/v) to yield fractions A - H. Fr.G was subjected to CC over a silica gel (100–200 mesh), eluting with a stepwise gradient of PE/EtOAc (from 15:1 to 1:1; that is, 15:1, 5:1, 3:1, and 1:1, v/v) to yield 4 fractions (Fr.G 1–4). Fr.G 3 was subjected to CC over a silica gel (200–300 mesh), eluting with a stepwise gradient of PE/EtOAc (from 10:1 to 3:1; that is, 10:1, 5:1, and 3:1, v/v) to yield 3 fractions (Fr.G 3-1-3). Fr.G 3-1 was purified using semi-preparative HPLC with MeOH/H_2_O (85:15, v/v) as the mobile phase to yield compound **1** (3.4 mg, *R*_t_ 15.2 min), **2** (4.2 mg, *R*_t_ 18.5 min), **8** (3.1 mg, *R*_t_ 22.3 min), **3** (5.2 mg, *R*_t_ 26.5 min), **4** (2.9 mg, *R*_t_ 50.5 min). Fr.G 3-2 was purified using semi-preparative HPLC of MeOH/H_2_O (90:10, v/v) as the mobile phase to yield compound **7** (3.0 mg, *R*_t_ 17.8 min). Fr.G 3-3 was purified using semi-preparative HPLC of MeOH/H_2_O (85:15, v/v) as the mobile phase to yield compound **5** (5.2 mg, *R*_t_ 17.8 min), **6** (4.7 mg, *R*_t_ 27.3 min).

### Spectroscopic Data

Nepetabrate E (**1**). Amorphous powder (MeOH−H_2_O, 90:10); ${\left[\alpha \right]}_{D}^{25}$ +153.0 (*c* 0.1, MeOH); IR (KBr) ʋ_max_ 3392, 1725 cm^−1^; ^1^H NMR (600 MHz, CDCl_3_) and ^13^C NMR (150 MHz, CDCl_3_) spectroscopic data, see Table 1; HRMS (ESI) *m/z* 305.1530 [M + Na]^+^ (calcd for C_19_H_22_O_2_Na 305.1517).

**TABLE 1:** NMR spectroscopic data for compounds **1**–**4** (600 MHz for ^1^H NMR and 150 MHz for ^13^C NMR).

	**1**		**2**		**3**		**4**	
position	*δ*_*C*_, type	*δ*_*H*_ (*J* in Hz)	*δ*_*C*_, type	*δ*_*H*_ (*J* in Hz)	*δ*_*C*_, type	*δ*_*H*_ (*J* in Hz)	*δ*_*C*_, type	*δ*_*H*_ (*J* in Hz)
1	33.0, CH_2_	2.94, 2.68, m	31.4, CH_2_	2.96, 2.88, m	21.5, CH_2_	2.00, 1.79, m	83.8, CH	3.90, m
2	34.1, CH_2_	2.79, 2.29, m	28.6, CH_2_	2.03, 1.92. m	30.3, CH_2_	2.98, m	69.4, CH	3.09, d (9.6)
3	199.6, C	-	80.8, CH	3.55, dd (4.8, 4.2)	73.1, C	-	44.8, CH_2_	1.54, m; 2.64, dd (4.2, 4.2)
4	130.4, C	-	36.9, C	-	151.3, C	-	38.6, C	-
5	159.8, C	-	50.8, CH	1.47, s	33.7, CH	2.85, m	49.9, CH	1.46, m
6	122.0, CH	6.81, d (10.2)	19.1, CH_2_	1.99, 1.69, m	32.3, CH_2_	1.89, 1.70, m	19.0, CH_2_	1.91, 1.81, m
7	135.8, CH	6.87, d (7.8)	29.9, CH_2_	1.45, 1.30, m	33.6, CH_2_	2.15, 1.97, m	29.9, CH_2_	3.09, 2.99, m
8	131.7, C	-	134.3, C	-	144.2, C	-	134.6, C	-
9	147.7, C	-	147.3, C	-	146.1, C	-	146.2, C	-
10	36.8, C	-	43.1, C	-	39.4, C	-	39.3, C	-
11	127.7, CH	7.19, dd, (1.8, 1.8)	124.6, CH	7.17, s	125.6, CH	7.23, d (8.4)	124.3, CH	7.18, d (8.4)
12	126.9, CH	7.09, d (1.8)	123.5, CH	7.17, s	124.2, CH	7.03, dd (1.8, 1.2)	124.3, CH	7.01, d (6.6)
13	142.5, C	-	143.9, C	-	134.9, C	-	146.1, C	-
14	124.0, CH	7.32, d (7.8)	126.2, CH	7.03, s	127.2, CH	6.94, s	127.1, CH	6.91, s
15	33.7, CH	2.68, m	21.2, CH	2.11, m	43.0, CH	2.85, m	33.6, CH	2.84, m
16	27.2, CH_3_	1.27, d (1.2)	22.6, CH_3_	1.32, s	24.2, CH_3_	1.24, s	28.8, CH_3_	1.10, s
17	24.1, CH_3_	1.25, d (1.2)	16.1, CH_3_	1.16, s	22.0, CH_3_	0.97, s	26.1, CH_3_	1.24, s
18	56.5, CH_2_	4.48, s	58.2, CH_2_	3.22, q (6.6, 7.2)	107.7, CH_2_	5.06, 4.84, s	16.7, CH_3_	0.94, s
19	-	-	64.4, CH_2_	4.34, m; 3.44, d (10.8)	28.1, CH_3_	0.90, s	24.2, CH_3_	1.23, s
20	30.4, CH_3_	1.44, s	26.1, CH_3_	1.16, s	68.6, CH_2_	3.97, 3.67, d (10.8)	24.1, CH_3_	1.22, s

Nepetabrate F (**2**). Amorphous powder (MeOH−H_2_O, 90:10); ${\left[\alpha \right]}_{D}^{25}$ +16.0 (*c* 0.1, MeOH); IR (KBr) ʋ_max_ 3365, 3563, 3645 cm^−1^; ^1^H NMR (600 MHz, CDCl_3_) and ^13^C NMR (150 MHz, CDCl_3_) spectroscopic data, see Table 1; HRMS (ESI) *m/z* 341.1815 [M + Na]^+^ (calcd for C_20_H_30_O_3_Na, 341.2093).

Nepetabrate G (**3**). Amorphous powder (MeOH−H_2_O, 90:10); ${\left[\alpha \right]}_{D}^{25}$ +24.0 (*c* 0.1, MeOH); IR (KBr) ʋ_max_ 3419, 3734, 1716 cm^−1^; ^1^H NMR (600 MHz, CDCl_3_) and ^13^C NMR (150 MHz, CDCl_3_) spectroscopic data, see Table 1; HRMS (ESI) *m/z* 323.2002 [M + Na]^+^ (calcd for C_20_H_28_O_2_Na, 323.1987).

Nepetabrate H (**4**). Amorphous powder (MeOH−H_2_O, 90:10); ${\left[\alpha \right]}_{D}^{25}$ +14.0 (*c* 0.1, MeOH); IR (KBr) ʋ_max_ 3303 cm^−1^; ^1^H NMR (600 MHz, CDCl_3_) and ^13^C NMR (150 MHz, CDCl_3_) spectroscopic data, see Table 1; HRMS (ESI) *m/z* 325.2157 [M + Na]^+^ (calcd for C_20_H_30_O_2_Na, 325.2143).

Nepetabrate I (**5**). Amorphous powder (MeOH−H_2_O, 85:15); ${\left[\alpha \right]}_{D}^{25}$ +40.0 (*c* 0.1, MeOH); IR (KBr) ʋ_max_ 2934, 1708 cm^−1^; ^1^H NMR (600 MHz, CDCl_3_) and ^13^C NMR (150 MHz, CDCl_3_) spectroscopic data, see Table 2; HRMS (ESI) *m/z* 367.2260 [M + Na]^+^ (calcd for C_22_H_32_O_3_Na, 367.2249).

**TABLE 2:** NMR spectroscopic data for compounds **5**–**8** (600 MHz for ^1^H NMR and 150 MHz for ^13^C NMR).

	**5**		**6**		**7**		**8**	
position	*δ*_*C*_, type	*δ*_*H*_ (*J* in Hz)	*δ*_*C*_, type	*δ*_*H*_ (*J* in Hz)	*δ*_*C*_, type	*δ*_*H*_ (*J* in Hz)	*δ*_*C*_, type	*δ*_*H*_ (*J* in Hz)
1	36.6, CH_2_	2.33, 1.58, m	37.1, CH_2_	2.36, 1.58, m	41.4, CH_2_	1.82, m; 1.77, d, 3.0)	29.9, CH_2_	1.27, m
2	31.2, CH_2_	2.95, 2.84, m	31.4, CH_2_	2.95, 2.86, m	20.0, CH_2_	1.63, 1.58, m	25.3, CH_2_	1.42, 1.08, m
3	82.7, CH	4.69, dd (4.8, 4.2)	78.9, CH	3.37, dd (4.8, 4.2)	37.5, CH_2_	2.20, 1.15, m	79.2, CH	3.21, dd (4.8, 4.2)
4	42.4, C	-	42.0, C	-	47.5, C	-	41.3, C	-
5	51.2, CH	1.55, m	51.1, CH	1.49, m	56.7, CH	1.77, d (2.4)	55.5, CH	0.69, m
6	19.6, CH_2_	2.00, 1.63, m	19.6, CH_2_	1.94, 1.80, m	25.9, CH_2_	2.35, 2.23, m	18.4, CH_2_	1.53, 1.23, m
7	28.1, CH_2_	1.94, 1.01, m	28.1, CH_2_	1.90, 1.58, m	44.5, CH_2_	2.43, m	33.5, CH_2_	1.41, 0.89, m
8	134.3, C	-	134.4, C	-	211.2, C	-	42.0, C	-
9	146.0, C	-	146.0, C	-	65.6, CH	3.10, s	48.7, CH	1.34, m
10	42.6, C	-	42.4, C	-	29.9, C	-	37.3, C	-
11	124.7, CH	7.17, d (7.8)	124.8, CH	7.17, d (7.8)	92.1, C	-	21.1, CH_2_	2.10, 1.51, m
12	124.2, CH	7.01, d (7.8)	124.2, CH	7.01, d (7.8)	27.6, CH_2_	2.30, 1.48, m	131.1, CH	7.54, m
13	145.7, C	-	145.7, C	-	29.3, CH_2_	2.56, m	129.0, C	-
14	126.8, CH	6.89, s	126.8, CH	6.89, s	177.6, C	-	40.7, C	-
15	33.4, CH	2.83, m	33.5, CH	2.83, m	37.8, CH	2.15, m	27.5, CH_2_	1.89, 1.69, m
16	21.5, CH_3_	2.11, s	21.2, CH_3_	2.08, s	29.7, CH_3_	1.33, s	27.2, CH_2_	2.02, 1.15, m
17	21.3, CH_3_	1.17, s	21.2, CH_3_	1.19, s	17.7, CH_3_	0.87, d (1.8)	39.0, C	-
18	63.8, CH_2_	4.27, d (6.6)	22.5, CH_3_	2.11, s	16.8, CH_3_	0.98, s	50.6, CH	1.33, m
		3.49, d (12.0)						
19	22.3, CH_3_	1.15, s	63.8, CH_2_	4.44, d (12.0)	181.8, C	-	41.9, CH	2.85, m
				4.25, d (12.0)				
20	169.9, C	-	171.2, C	-	16.3, CH_3_	1.25, s	84.3, C	-
21	24.0, CH_3_	1.22, s	24.0, CH_3_	1.22, s	-	-	72.3, CH	3.79, m
22	25.8, CH_3_	1.21, s	25.5, CH_3_	1.21, s	-	-	34.1, CH_2_	2.35, 1.89, m
23	-	-	-	-	-	-	176.6, C	-
24	-	-	-	-	-	-	15.5, CH_3_	0.80, s
25	-	-	-	-	-	-	14.4, CH_3_	0.96, s
26	-	-	-	-	-	-	15.9, CH_3_	0.69, s
27	-	-	-	-	-	-	28.2, CH_3_	1.00, d (7.2)
28	-	-	-	-	-	-	21.3, CH_3_	1.04, s
29	-	-	-	-	-	-	16.5, CH_3_	0.89, d (2.4)
30	-	-	-	-	-	-	18.8, CH_3_	1.20, s

Nepetabrate J (**6**). Amorphous powder (MeOH−H_2_O, 85:15); ${\left[\alpha \right]}_{D}^{25}$ +425.93 (*c* 0.1, MeOH); IR (KBr) ʋ_max_ 2934, 1708 cm^−1^; ^1^H NMR (600 MHz, CDCl_3_) and ^13^C NMR (150 MHz, CDCl_3_) spectroscopic data, see Table 2; HRMS (ESI) *m/z* 367.2256 [M + Na]^+^ (calcd for C_22_H_32_O_3_Na, 367.2249).

Nepetabrate K (**7**). Amorphous powder (MeOH−H_2_O, 90:10); ${\left[\alpha \right]}_{D}^{25}$ -5.0 (*c* 0.1, MeOH); IR (KBr) ʋ_max_ 1607, 1656, 1708 cm^−1^; ^1^H NMR (600 MHz, CDCl_3_) and ^13^C NMR (150 MHz, CDCl_3_) spectroscopic data, see Table 2; HRMS (ESI) *m/z* 373.1991 [M + Na]^+^ (calcd for C_20_H_30_O_5_Na, 373.2002).

Vanguerolactone (**8**). Amorphous powder (MeOH−H_2_O, 85:15); ${\left[\alpha \right]}_{D}^{25}$ +10 (*c* 0.1, MeOH); IR (KBr) ʋ_max_ 3420, 1729 cm^−1^; ^1^H NMR (600 MHz, CDCl_3_) and ^13^C NMR (150 MHz, CDCl_3_) spectroscopic data, see Table 2; HRMS (ESI) *m/z* 493.3272 [M + Na]^+^ (calcd for C_30_H_46_O_4_Na, 493.3294).

### ECD Computational Methods

All calculations were performed using Gaussian 09 software (Frisch et al., 2013). The stable conformers subjected to ECD calculation were optimized using the time-dependent density functional theory (TDDFT) method at the B3LYP/6-31G(d) level of theory, and solvent effects of the MeOH solution were evaluated at the same DFT level using the SCRF/PCM procedure (Shi et al., 2019). The final calculated ECD spectra were obtained according to the Boltzmann-calculated contribution of each conformer.

### RAW 264.7 Macrophage Viability Test

The MTT colorimetric method was used to detect the effect of compounds **1–8** on the viability of RAW 264.7 macrophages. The RAW 264.7 macrophages in the logarithmic growth phase were digested with trypsin to prepare a single-cell suspension, which was seeded in a 96-well plate at a density of 1 × 10^4^ cells per well and cultured in a 5% CO_2_ incubator for 24 h at 37°C, before discarding the supernatant. The blank control group was cultured with 10% FBS-containing DMEM, and the drug group was treated with aqueous solutions of compounds **1–8**, with six replicate wells for each concentration. Incubation was continued in 5% CO_2_ at 37°C. After 24 h of incubation, 10 µl of 5 mg/ml MTT was added to each well. The culture solution was removed after culturing for 4 h. Then, 100 µL of dimethyl sulfoxide (DMSO) was added to each well, before shaking for 10 min to achieve complete dissolution. The optical density (OD) was measured at 492 nm using a microplate reader to calculate cell viability.

### Anti-Inflammation Assay

The anti-inflammatory activity of the isolated compounds was evaluated in LPS-stimulated RAW 264.7 macrophages using the MTT colorimetric method. The RAW 264.7 macrophages were seeded in 96-well plates at a density of 1 × 104 cells per well for 24 h, followed by treatment with different extracts of identical purity for another 24 h. The compounds were dissolved in DMSO and diluted appropriately just before cell treatments. Cells were incubated with the extract at indicated concentrations, with DMSO not exceeding 0.1% in all experiments. The cells were cultured in DMEM with 10% FBS and antibiotics (100 U/mL penicillin and 100 μg/ml streptomycin) at 37°C with 5% CO2. NO release was measured as an indicator of the nitrite concentration.

## Results and Discussion

### Structure Elucidation of Compounds

**FIGURE 2:**
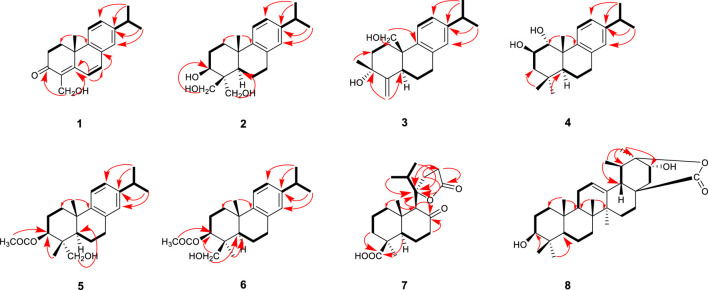
Key HMBC (arrows) and^1^H−^1^H COSY (bold lines) correlations for compounds **1–8**.

**FIGURE 3:**
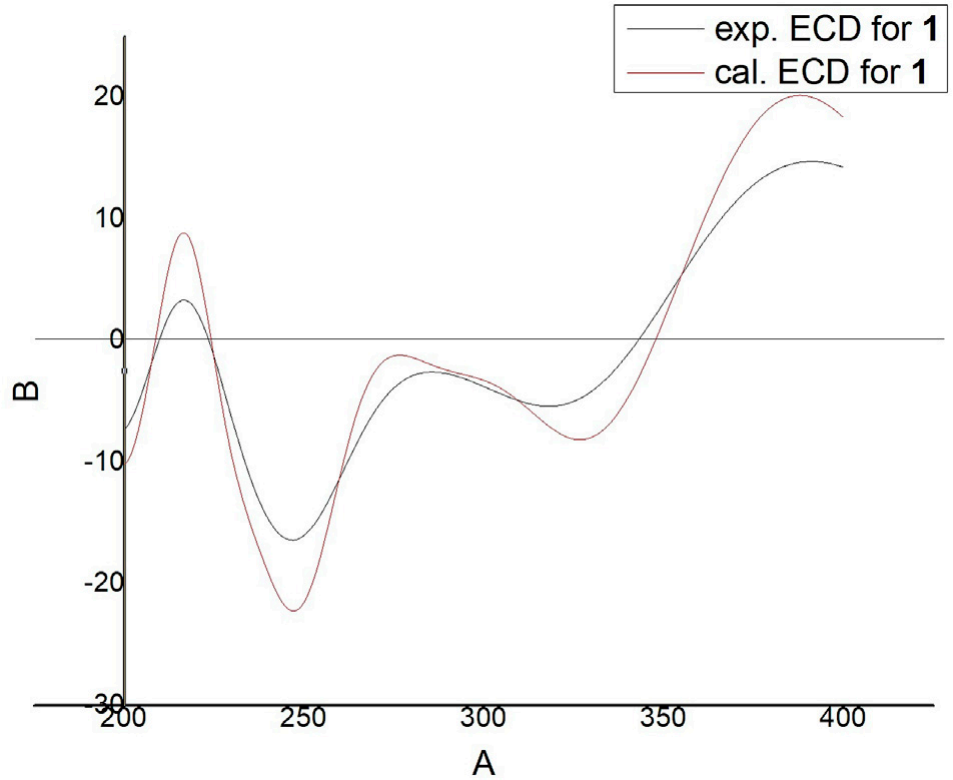
Experimental and calculated ECD spectra of Compound **1**.

Compound **1** was isolated as an amorphous powder. Its molecular formula was assigned as C_19_H_22_O_2_ based on the ^13^C NMR spectroscopic data (Table 1) and the positive HRMS (ESI) ion peak at *m/z* 305.1530 [M + Na]^+^ (calculated C_19_H_22_O_2_Na 305.1517). The IR spectrum of **1** showed absorptions of hydroxyl (3392 cm^−1^) and keto carbonyl (1725 cm^−1^) groups. In the ^1^H-NMR spectrum, compound **1** showed peaks for three aromatic protons at *δ*_H_ 7.32 (1H, d, *J* = 7.8 Hz), 7.19 (1H, dd, *J* = 7.8, 1.8 Hz), and 7.09 (1H, d, *J* = 1.8 Hz), which suggests the presence of a benzene moiety. Three methyl protons at *δ*_H_ 1.27 (3H, d, *J* = 1.2 Hz), 1.25 (3H, d, *J* = 1.2 Hz), 1.44 (3H, s) indicate the basic diterpenoid skeleton (Zhang et al., 2013). The downfield proton signal at *δ*_H_ 4.48 (2H, s) suggests the presence of -CH_2_OH group in the structure, which is in accordance with the ^13^C-NMR signal showing at *δ*_C_ 56.5. The^13^C-NMR spectrum revealed six aromatic carbon signals at *δ*_C_ 131.7, 147.7, 127.7, 126.9, 142.6, 124.0 and one carbonyl carbon signal at *δ*_C_ 199.6. Aside from these above carbons, the ^13^C-NMR also showed three methyl signals at *δ*_C_ 27.2, 24.2 and 30.4, three methylene signals at *δ*_C_ 33.0, 34.1 and 56.5, three methine signals at *δ*_C_ 122.0, 135.8 and 33.7, and three quartus carbon signals at *δ*_C_ 130.4, 159.8, 36.8. The proton signals were assigned to the corresponding carbons through direct ^1^H and ^13^C correlations in the HSQC spectrum. From the ^1^H—^1^H COSY analysis, four substructures were established as H-1/H-2, H-6/H-7, H-11/H-12 and H-15/H-16/H-17 (Figure 2), suggesting that compound **1** has an abietane diterpene skeleton (Zhang et al., 2013). In the HMBC spectrum, the correlations from H-18 (*δ*_H_ 4.48) to C-3 (*δ*_C_ 199.6) and C-5 (*δ*_C_ 159.8), from H-6 (*δ*_H_ 6.81) to C-5 (*δ*_C_ 159.8) and C-7 (*δ*_C_ 135.8) and from H-7 (*δ*_H_ 6.87) to C-8 (*δ*_C_ 131.7) implied the α, β-unsaturated keton at C-3/C-4/C-5 and the double bond at C-6/C-7, as well as substitutions of its hydroxymethyl group (Figure 2). Thus, the planar structure of compound **1** was fully elucidated. Considering the identical biosynthetic relationship of abietane diterpenoids, the absolute configuration of **1** can be inferred as 10*S*. The ECD spectra were calculated using density functional theory (DFT) at the APFD/6-311 + g (2d, p) level to further support the deduction, the absolute configuration of C-10 was assigned as *S* on the basis of a comparison of its experimental and calculated CD curves (Figure 3). As a result, the structure of compound **1** was determined as shown and given the trivial name nepetabrate E (Zhang et al., 2021). Compound **1** was representative of a new degraded carbon abietane diterpene which was not common in the natural products.

**FIGURE 4:**
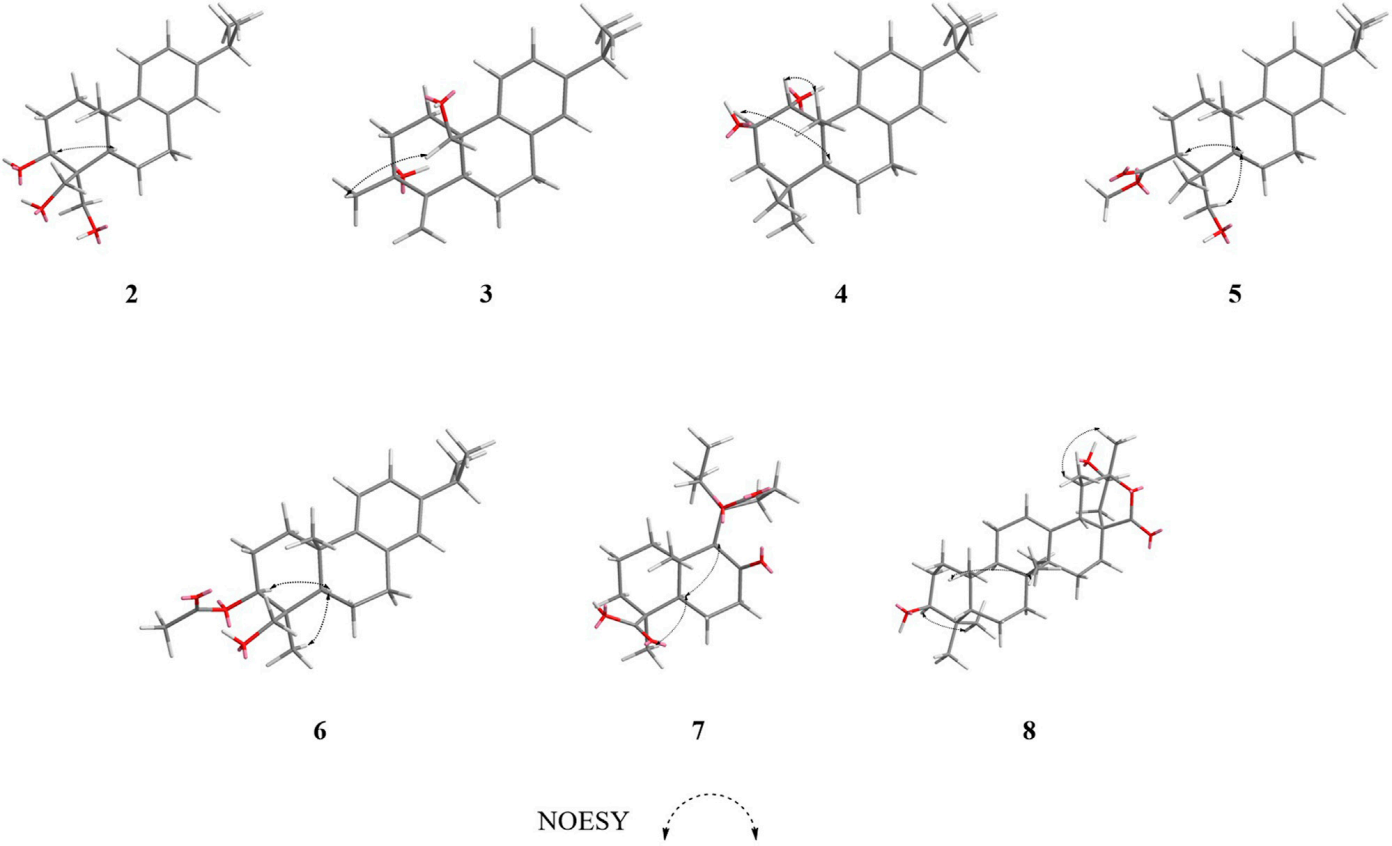
Key NOESY correlations of compounds **2–8**.

Compound **2** was obtained as an amorphous powder with a molecular formula of C_20_H_20_O_3_ based on the HRMS (ESI) protonated molecular ion peak at *m/z* 341.1815 [M + Na]^+^ (calcd for C_20_H_30_O_3_Na, 341.2093). The IR spectrum of **2** showed absorptions of hydroxyl (3365, 3563, 3645 cm^−1^) groups. Through the ^1^H- and ^13^C-NMR spectra (Table 1), we inferred that the basic mother nucleus of compound **2** was an abietane diterpene, which was further confirmed by the ^1^H–^1^H COSY and HMBC spectra. The ^1^H- and ^13^C-NMR spectroscopic data of **2** were similar to those of compound **1**, except for the additional hydroxymethyl group at *δ*_C_ 64.4 and hydroxyl group at *δ*_C_ 80.8, indicating that compound **2** is an analogue of compound **1**. One hydroxyl group placed at C-3 and the saturated carbon C-4/C-5/C-6/C-7 led to the higher field chemical shift of C-3/C-4/C-5/C-6/C-7 (*δ*_C_ 80.8, 36.9, 50.8, 19.1, 29.9, respectively) compared with **1**, which suggesting the ketone carbonyl and double bond in **1** were hydrogenated in **2,** as confirmed by HMBC correlations (Figure 2). The relative configuration of compound **2** was established by analysis of its NOESY data (Figure 4). The key NOE correlations of H-3 and H-5 supported the β-orientations of hydroxyl group at C-3. Combined with the experimental and calculated CD curves (Supplementary Figure S2.8), the absolute configuration of compound **2** was identified as established and given the trivial name nepetabrate F.

Compound **3**, purified as an amorphous powder, has a molecular formula of C_20_H_28_O_2_, deduced from the HRMS (ESI) protonated molecular ion peak at *m/z* 323.2002 [M + Na]^+^ (calculated C_20_H_28_O_2_Na, 323.1987). The IR spectrum of **3** showed absorptions of hydroxyl (3419, 3734 cm^−1^) and double bond (1716 cm^−1^) groups. Compound **3** was also an abietane diterpene, which was further confirmed by the ^1^H–^1^H COSY and HMBC spectra. The ^1^H- and ^13^C-NMR spectroscopic data (Table 1) of **3** were similar to those of compound **2**, except for the additional double bond at *δ*_C_ 151.3, 107.7, and the disappearance of one hydroxymethyl group indicating that compound **3** is an analogue of compound **2**. One double bond placed at C-4 led to the downfield chemical shift of C-4 (*δ*_C_ 151.3), as confirmed by HMBC correlations from H-18 (*δ*_H_ 5.06, 4.84) to *δ*_C_ 73.1 (C-3), *δ*_C_ 33.7 (C-5) (Figure 2). Meanwhile, one methyl group was transferred to C-3 based on the HMBC correlations from H-19 (*δ*_H_ 0.90) to *δ*_C_ 73.1 (C-3), *δ*_C_ 151.3 (C-4). Furthermore, the methyl group at C-10 in **2** was oxygenated to hydroxymethyl group in **3** according to the HMBC correlations from H-20 (*δ*_H_ 3.97, 3.67) to *δ*_C_ 39.4 (C-10). The relative configuration of compound **3** was established by analysis of its NOESY data (Figure 3). The key NOE correlations between H-19 and H-20 supported the β-orientation of hydroxyl group at C-3. Combined with the experimental and calculated CD curves (Supplementary Figure S3.8), the absolute configuration of compound **3** was identified as established and given the trivial name nepetabrate G.

Compound **4** was obtained as an amorphous powder with its molecular formula assigned as C_20_H_30_O_2_ according to the HRMS (ESI) protonated molecular ion peak at *m/z* 325.2157 [M + Na]^+^ (calculated C_20_H_30_O_2_Na, 325.2143). The IR spectrum of **4** showed absorptions of hydroxyl (3303 cm^−1^) group. The ^1^H- and ^13^C-NMR spectroscopic data (Table 1) of **4** were similar to those of compound **2**, except for the presence of oxygenated methine carbon signals at *δ*_C_ 69.4, 83.8, indicating that compound **4** is an analogue of compound **2**. Two hydroxyl groups placed at C-1 and C-2 led to the downfield chemical shift of C-1/C-2 (*δ*_C_ 83.8, 69.4, respectively), as confirmed by HMBC correlations from H-1 (*δ*_H_ 3.90) to C-2 (*δ*_C_ 69.4) and from H-2 (*δ*_H_ 3.09) to C-1 (*δ*_C_ 83.8) (Figure 2). The two methyl signals at *δ*_H_ 0.94 (H-18) and *δ*_H_ 1.23 (H-19) implied the hydroxymethyl groups in **2** were reduced in 4. The relative configuration of compound **4** was established by its NOESY spectrum (Figure 3) and the coupling constants. The key NOE correlations between H-1 and H-20, H-2 and H-5 supported the α-orientation of hydroxyl group at C-1 and β-orientation at C-2. The large coupling constant between H-1 and H-2 (*J* = 9.6 Hz) also supported their antiperiplannar relationship. Combined with the experimental and calculated CD curves (Supplementary Figure S4.8), the absolute configuration of compound **4** was identified as established and given the trivial name nepetabrate H.

Compound **5** was given as an amorphous powder. The positive HRMS (ESI) showed a protonated molecular ion peak at *m/z* 367.2260 [M + Na]^+^ (calculated C_22_H_32_O_3_Na 367.2249), indicating its molecular formula of C_22_H_32_O_3._ The IR spectrum of **5** showed absorptions of hydroxyl (2934 cm^−1^) and ester carbonyl (1708 cm^−1^) groups. The ^1^H- and ^13^C-NMR spectroscopic data (Table 2) of **5** were similar to those of compound **2**, except for the additional acetyl group at *δ*_C_ 82.7, and the disappearance of one hydoxymethyl group, suggesting that compound **5** is an analogue of compound **2**. Meanwhile, three oxygenated protons at *δ*_H_ 4.69 (1H, dd, *J* = 12.0, 4.8 Hz), 4.27 (1H, d, *J* = 12.0 Hz), and 3.49 (1H, d, *J* = 12.0 Hz) suggest the presence of –OCH_2_– and –OCH– groups in the structure, which is in accordance with the ^13^C-NMR spectrum showing signals at *δ*_C_ 82.7, 63.8. In the HMBC spectrum, the correlations from *δ*_H_ 4.69 (1H, dd, *J* = 12.0, 4.8 Hz) to *δ*_C_ 169.9 indicate that the acetoxyl group is attached to C-3. Furthermore, the HMBC correlations from *δ*_H_ 4.27 (1H, d, *J* = 12.0 Hz) and 3.49 (1H, d, *J* = 12.0 Hz) to C-4 (*δ*_C_ 42.4) and C-5 (*δ*_C_ 51.2) indicate that the hydroxylmethyl group at C-4 (Figure 2). In the NOESY spectrum (Figure 3), the enhancement between H-3 and H-5, H-18 and H-5 suggests the β-orientation of the acetoxyl group at C-3. Considering the identical biosynthetic relationship of abietane diterpenoids, the absolute configuration of **5** can be inferred as 3*S*, 4*R*, 4*R*, 10*S*. The ECD spectra were calculated using density functional theory (DFT) at the APFD/6-311 + g (2d, p) level to further support the deduction (Supplementary Figure S5.8). As a result, the structure of compound **5** was determined as shown and given the trivial name nepetabrate I.

Compound **6** was isolated as an amorphous powder. Its molecular formula, C_22_H_32_O_3_, was determined by the HRMS (ESI) protonated molecular ion peak at *m/z* 367.2256 [M + Na]^+^ (calculated C_22_H_32_O_3_Na, 367.2249). The IR spectrum of **6** showed absorptions of hydroxyl (2934 cm^−1^) and ester carbonyl (1708 cm^−1^) groups. The ^1^H- and ^13^C-NMR spectroscopic data (Table 2) of **6** were similar to those of compound **5**, except for the differential chemical shifts of hydroxylmethyl group at *δ*_C_ 51.1 compared with *δ*_C_ 63.2 in **5** which implied the diversity configurations of hydroxymehtyl group. In the NOESY spectrum (Figure 3), the enhancement between H-19 and H-20 indicate the β-orientation of hydroxylmethyl group at C-4, which is contrary to the compound **5**. Absolute configuration can be confirmed by experimental and calculated CD curves (Supplementary Figure S6.8). Thus, the structure of compound **6** was determined as shown and given the trivial name nepetabrate J.

**SCHEME 1:**
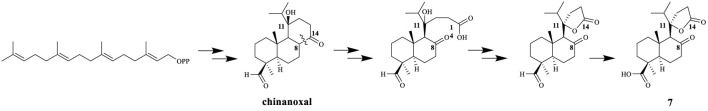
Postulated Biosynthesis Pathways of compound **7**.

Compound **7** was isolated as an amorphous powder. Its molecular formula was established as C_20_H_30_O_5_, based on the HRMS (ESI) protonated molecular ion peak at *m/z* 373.1991 [M + Na]^+^ (calculated C_20_H_30_O_5_Na, 373.2002) and was supported by the ^13^C NMR spectroscopic data. The IR spectrum of **7** showed absorptions of keto carbonyl (1607 cm^−1^), carboxy carbonyl (1656 cm^−1^) and ester carbonyl (1708 cm^−1^) groups. In the ^1^H-NMR spectrum (Table 2), compound **7** showed four methyl protons at *δ*_H_ 1.33 (3H, s), 0.87 (3H, d, *J* = 1.8 Hz), 0.98 (3H, s), and 1.25 (3H, s), indicated that there may be a terpene-like skeleton. The^13^C-NMR spectrum showed three downfield carbon signals at *δ*_C_ 211.2, 177.6 and 181.8, which demonstrated three carbonyl carbon signals as deduced by IR spectrum. In addition to these carbon signals, the ^13^C-NMR also showed four methyl signals at *δ*_C_ 29.7, 17.7, 16.8 and 16.3; seven methylene signals at *δ*_C_ 41.4, 20.0, 37.5, 25.9, 44.5, 27.6 and 29.3; three methine signals at *δ*_C_ 56.7, 65.6 and 37.8; three quartus carbon signals at *δ*_C_ 47.5, 29.9 and 92.1. 2D NMR experiments (COSY, HMQC, and HMBC) enabled the full assignments of all proton and carbon atoms. In the HMBC spectrum, the correlations from *δ*_H_ 2.43 (2H, m, H-7) and *δ*_H_ 3.10 (1H, s, H-9) to *δ*_C_ 211.2 indicated that the keto carbonyl group was attached to C-8; from *δ*_H_ 3.10 (1H, s, H-9), *δ*_H_ 2.30 (1H, m, H-12) and *δ*_H_ 2.15 (1H, m, H-15) to *δ*_C_ 92.1(C-11), from *δ*_H_ 2.30 (1H, m, H-12) to *δ*_C_ 29.3 (C-13) and from *δ*_H_ 2.56 (2H, m, H-13) to *δ*_C_ 177.6 (C-14) demonstrated that the four carbon atoms C-11/12/13/14 made up a five-membered lactone ring; from *δ*_H_ 1.15 (1H, m, H-3) and 1.77 (1H, d, *J* = 2.4 Hz, H-5) to *δ*_C_ 181.8 (C-19) indicated the carboxyl group was attached to C-4 (Figure 2). Of particular significance were the long-range correlations from C-15 (*δ*_C_ 37.8) to H-9 (*δ*_H_ 3.10) and H-12 (*δ*_H_ 2.30), as well as the correlations from C-11 (*δ*_C_ 92.1) to H-9 (*δ*_H_ 3.10), H-12 (*δ*_H_ 2.30), H-16 (*δ*_H_ 0.87), and H-17 (*δ*_H_ 0.87). These data confirmed the location of the isopropyl group at C-11. The NOE enhancements H-5 and H_3_-28, H-5 and H-9 displayed the β-orientation of carboxy group at C-4 and rearranged ring at C-9. The absolute configuration of 4*S*, 5*R*, 9*S*, 10*S*, 11*R* were assigned on the basis of a comparison of its experimental and calculated CD curves (Supplementary Figure S7.8). From all the above data, compound **7** was established as shown and named nepetabrate K. Compound **7** was the first example of norabietane-type diterpenoid skeleton containing five-membered lactone from the genus *Nepeta*. The most plausible biosynthesis pathway for the formation of compound **7** from chinanoxal (Fang et al., 1993) was the oxidative cleavage between C-8 and C-14 to the diketones and then esterification to the novel secoditerpenal **7** (Lee and Cheng, 2001) (Scheme 1).

Compound **8** was isolated as an amorphous powder. The HRMS (ESI) indicated a precise [M + Na]^+^ ion at *m/z* 493.3272 (calculated C_30_H_46_O_4_Na, 493.3294), together with ^13^C NMR spectroscopic data indicating an empirical molecular formula of C_30_H_46_O_4_. The IR spectrum of **8** showed absorptions of hydroxyl (3420 cm^−1^) and ester carbonyl (1729 cm^−1^) groups. In the ^1^H-NMR spectrum (Table 2), compound **8** showed seven highfield methyl signals at *δ*_H_ 0.80 (3H, s), 0.96 (3H, s), 0.69 (3H, s), 1.00 (3H, d, *J* = 7.2 Hz), 1.04 (3H, s), 0.89 (3H, d, *J* = 2.4 Hz) and 1.20 (3H, s). In the ^13^C-NMR spectrum, compound **8** showed 30 carbon signals including one double bond at *δ*_C_131.1, 129.0 and one ester carbonyl signal at *δ*_C_ 176.6 suggesting its triterpenoid scaffold. The proton signals were assigned to the corresponding carbons through direct ^1^H and ^13^C correlations in the HSQC spectrum. The data above suggested that compound **8** has an ursane triterpene skeleton (Chen et al., 2008). In the HMBC spectrum, the correlations from H-30 (*δ*_H_ 1.20) to *δ*_C_ 79.2 (C-3) and H-24 (*δ*_H_ 0.80) to *δ*_C_ 72.3 (C-21) as well as the molecular formula above implied hydroxyl groups at C-3 and C-21. Meanwhile, the upfield carbonyl group at *δ*_C_ 176.6 (C-23) compared with the reported ones (Manzoor-i-Khuda and Habermehl, 1979) exhibited the formation of ester bond between C-23 and C-20 as confirmed by HMBC correlations together with the unsaturated degree (Figure 2). The NOE enhancements between H-3 and H-5, H-18 and H-21 suggested the β-orientaiton of hydroxyl group at C-3, and α-orientaiton of hydroxyl unit at C-21. The weak CD curve of compound **8** made assignment of the absolute configurations unreliable. Considering the basic scaffold and biosynthetic pathway of ursane-type triterpenoid, compound **8** was established as shown and named vanguerolactone (Manzoor-i-Khuda and Habermehl, 1979).

### Bioactive Activity

**FIGURE 5:**
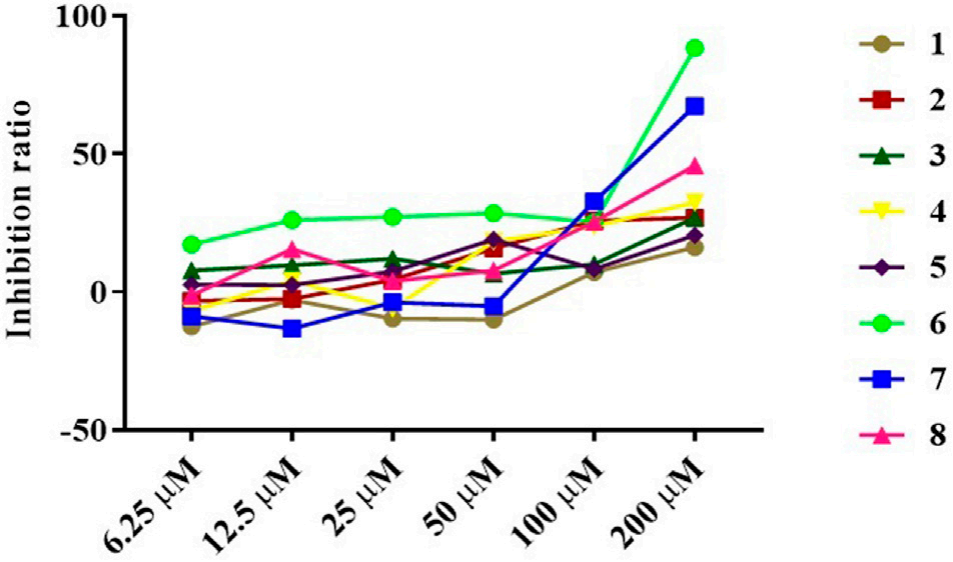
RAW 264.7 macrophage viability test of the isolated compounds.

**FIGURE 6:**
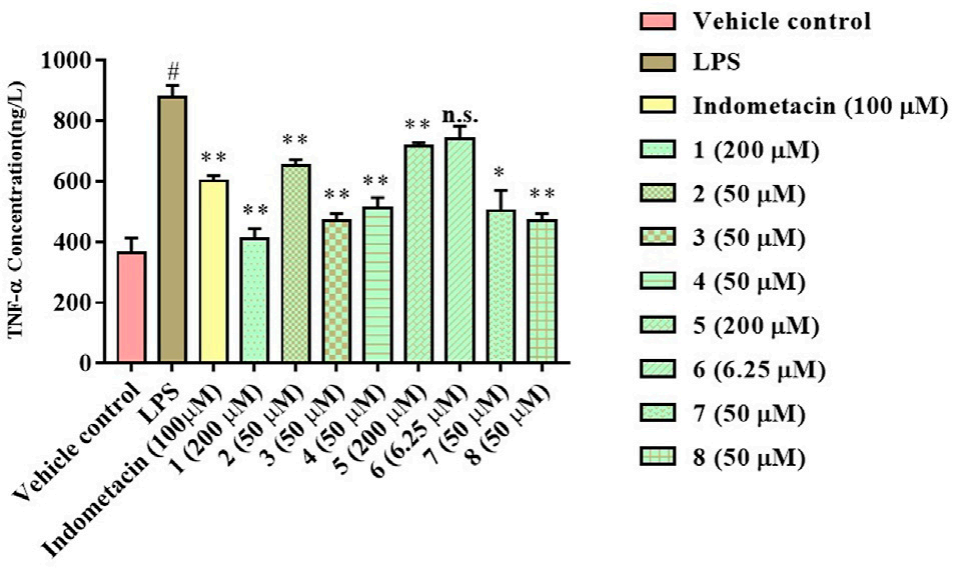
Anti-inflammation assay of the isolated compounds (^#^ *p* < 0.05 vs the Vehicle control; **p < 0.01, *p < 0.05, n.s. indicates no significant vs the LPS model).

The anti-inflammatory activity of the isolated compounds was evaluated in lipopolysaccharide-stimulated RAW 264.7 macrophages using the MTT colorimetric method, RAW 264.7 macrophage viability test results show that compounds **2**, **4**, **6**-**8** had a certain inhibitory effect on the viability of RAW264.7 cells at lower concentrations (≤50 μM), especially compound **6**, which inhibited the viability of RAW264.7 cells at 6.25 μM, while compound **1**, **3**, and **5** exhibited mild effect on RAW264.7 cell viability at higher concentrations (≥100 μM), as indicated in Figure 5 and Table 3. Compound concentrations that did not significantly affect cell viability were next selected for subsequent screening for anti-inflammatory activity. The inflammatory factor TNF-α was used as the detection index to screen the anti-inflammatory activity of compounds **1**-**8**. The results of anti-inflammatory activity screening showed that compared with the LPS model group, except for compound **6**, the other seven compounds were all significantly down-regulation the typical inflammatory factor TNF-α at the specific concentration, as indicated in Figure 6. It is interesting that the chemical structures of compounds **5** and **6** are highly similar, but their effects on anti-inflammatory are very different, which may be related to the stereo-configuration of -CH_2_OH at C-4. Compound **3** and **8** showed the most potent anti-inflammatory, compound **2, 4**, and **7** showed moderate anti-inflammatory, while compound **1** and **5** showed poor anti-inflammatory activities among all the isolates in Figure 6, we can infer that the C-3 position of the compounds has no group substitution or is substituted with an electron donating group such as hydroxyl, and the anti-inflammatory activity is better; while the C-3 position has an electron withdrawing group such as carbonyl or acetyl substitution, the anti-inflammatory activity is poor, the structure-activity relationship is consistent with the results reported in the previous literature(Liu et al., 2016; Nie et al., 2021). From the results, we can infer that abietane-type diterpenoids were the material basis for *N. bracteata* Benth to exert anti-inflammatory effect in clinic.

**TABLE 3:** RAW 264.7 macrophage viability test of the isolated compounds.

Concentration (µM)	**1**	**2**	**3**	**4**	**5**	**6**	**7**	**8**
	Inhibition ratio							
6.25	−12.4066	−3.13901	7.847534	−6.85102	2.640757	17.21475	−8.71948	−1.22073
12.5	−2.86497	−2.41654	9.666168	4.185351	2.516193	26.10862	−13.1789	15.59542
25	−9.59143	4.459392	12.0578	−5.90433	7.473842	27.20478	−3.66218	3.91131
50	−9.9153	15.76981	6.676632	18.31091	19.00847	28.52516	−5.03239	7.847534
100	7.149975	25.75984	10.08969	23.79173	8.320877	25.33632	32.85999	25.38615
200	16.16841	26.93074	26.90583	32.4863	20.55306	88.44046	67.46388	45.86447
IC_50_(µmol/L)	4419.846	1304.963	2652.011	607.169	6785.538	59.403	136.059	292.284

## Conclusion

In summary, seven new abietane-type diterpenoids (**1–7**), along with a new ursane-type triterpenoid (**8**), were isolated from the extracts of *N. bracteata* Benth. The structures of all compounds were elucidated by extensive spectroscopic analysis, including 1D, 2D NMR, and HR-MS(ESI), in combination with ECD spectra. Compound **1** was representative of a new degraded carbon abietane diterpene which was not common in the natural products, and compound **7** was the first example of norabietane-type diterpenoid skeleton containing five-membered lactone from the genus *Nepeta*. The results of anti-inflammatory activity screening showed that compared with the LPS model group, except for compound **6**, the other seven compounds were all remarkable down-regulation the content of TNF-α at the specific concentration.

## Data Availability

The original contributions presented in the study are included in the article Supplementary Material, further inquiries can be directed to the corresponding authors.
