# Supplementary material for: Abietane-Type Diterpenoids From Nepeta bracteata Benth. and Their Anti-Inflammatory Activity
Source: Front Chem. 2022 Jul 4;10:944972. doi: 10.3389/fchem.2022.944972 (PMC9289214; doi:10.3389/fchem.2022.944972)
Supplement: Supplementary file 1 [file DataSheet1.pdf]

Supplementary Material

Figure S1.1 ^{1}H NMR spectrum of compound **1** (600 MHz, $CDCl_3$)

Figure S1.2 ^{13}C APT spectrum of compound **1** (150 MHz, $CDCl_3$)

Figure S1.3 HSQC spectrum of compound **1**

Figure S1.4 HMBC spectrum of compound **1**

Figure S1.5 ^{1}H-^{1}H COSY spectrum of compound **1**

Figure S1.6 NOESY spectrum of compound **1**

Figure S1.7 Positive ion HRESIMS of compound **1**

Figure S1.8 Experimental and calculated ECD spectra of compound **1**

Figure S2.1 ^{1}H NMR spectrum of compound **2** (600 MHz, $CDCl_3$)

Figure S2.2 ^{13}C APT spectrum of compound **2** (150 MHz, $CDCl_3$)

Figure S2.3 HSQC spectrum of compound **2**

Figure S2.4 HMBC spectrum of compound **2**

Figure S2.5 ^{1}H-^{1}H COSY spectrum of compound **2**

Figure S2.6 NOESY spectrum of compound **2**

Figure S2.7 Positive ion HRESIMS of compound **2**

Figure S2.8 Experimental and calculated ECD spectra of compound **2**

Figure S3.1 ^{1}H NMR spectrum of compound **3** (600 MHz, $CDCl_3$)

Figure S3.2 ^{13}C APT spectrum of compound **3** (150 MHz, $CDCl_3$)

Figure S3.3 HSQC spectrum of compound **3**

Figure S3.4 HMBC spectrum of compound **3**

Figure S3.5 ^{1}H-^{1}H COSY spectrum of compound **3**

Figure S3.6 NOESY spectrum of compound **3**

Figure S3.7 Positive ion HRESIMS of compound **3**

Figure S3.8 Experimental and calculated ECD spectra of compound **3**

Figure S4.1 ^{1}H NMR spectrum of compound **4** (600 MHz, $CDCl_3$)

Figure S4.2 ^{13}C APT spectrum of compound **4** (150 MHz, $CDCl_3$)

Figure S4.3 HSQC spectrum of compound **4**

Figure S4.4 HMBC spectrum of compound **4**

Figure S4.5 ^{1}H-^{1}H COSY spectrum of compound **4**

Figure S4.6 NOESY spectrum of compound **4**

Figure S4.7 Positive ion HRESIMS of compound **4**

Figure S4.8 Experimental and calculated ECD spectra of compound **4**

Figure S5.1 ^{1}H NMR spectrum of compound **5** (600 MHz, $CDCl_3$)

Figure S5.2 ^{13}C APT spectrum of compound **5** (150 MHz, $CDCl_3$)

Figure S5.3 HSQC spectrum of compound **5**

Figure S5.4 HMBC spectrum of compound **5**

Figure S5.5 ^{1}H-^{1}H COSY spectrum of compound **5**

Figure S5.6 NOESY spectrum of compound **5**

Figure S5.7 Positive ion HRESIMS of compound **5**

Figure S5.8 Experimental and calculated ECD spectra of compound **5**

Figure S6.1 ^{1}H NMR spectrum of compound **6** (600 MHz, $CDCl_3$)

Figure S6.2 ^{13}C APT spectrum of compound **6** (150 MHz, $CDCl_3$)

Figure S6.3 HSQC spectrum of compound **6**

Figure S6.4 HMBC spectrum of compound **6**

Figure S6.5 ^{1}H-^{1}H COSY spectrum of compound **6**

Figure S6.6 NOESY spectrum of compound **6**

Figure S6.7 Positive ion HRESIMS of **6**

Figure S6.8 Experimental and calculated ECD spectra of compound **6**

Figure S7.1 ^{1}H NMR spectrum of compound **7** (600 MHz, $CDCl_3$)

Figure S7.2 ^{13}C APT spectrum of compound **7** (150 MHz, $CDCl_3$)

Figure S7.3 HSQC spectrum of compound **7**

Figure S7.4 HMBC spectrum of compound **7**

Figure S7.5 ^{1}H-^{1}H COSY spectrum of compound **7**

Figure S7.6 NOESY spectrum of compound **7**

Figure S7.7 Positive ion HRESIMS of compound **7**

Figure S7.8 Experimental and calculated ECD spectra of compound **7**

Figure S8.1 ^{1}H NMR spectrum of compound **8** (600 MHz, $CDCl_3$)

Figure S8.2 ^{13}C APT spectrum of compound **8** (150 MHz, $CDCl_3$)

Figure S8.3 HSQC spectrum of compound **8**

Figure S8.4 HMBC spectrum of compound **8**

Figure S8.5 ^{1}H-^{1}H COSY spectrum of compound **8**

Figure S8.6 NOESY spectrum of compound **8**

Figure S8.7 Positive ion HRESIMS of compound **8**

Figure S8.8 Experimental and calculated ECD spectra of compound **8**

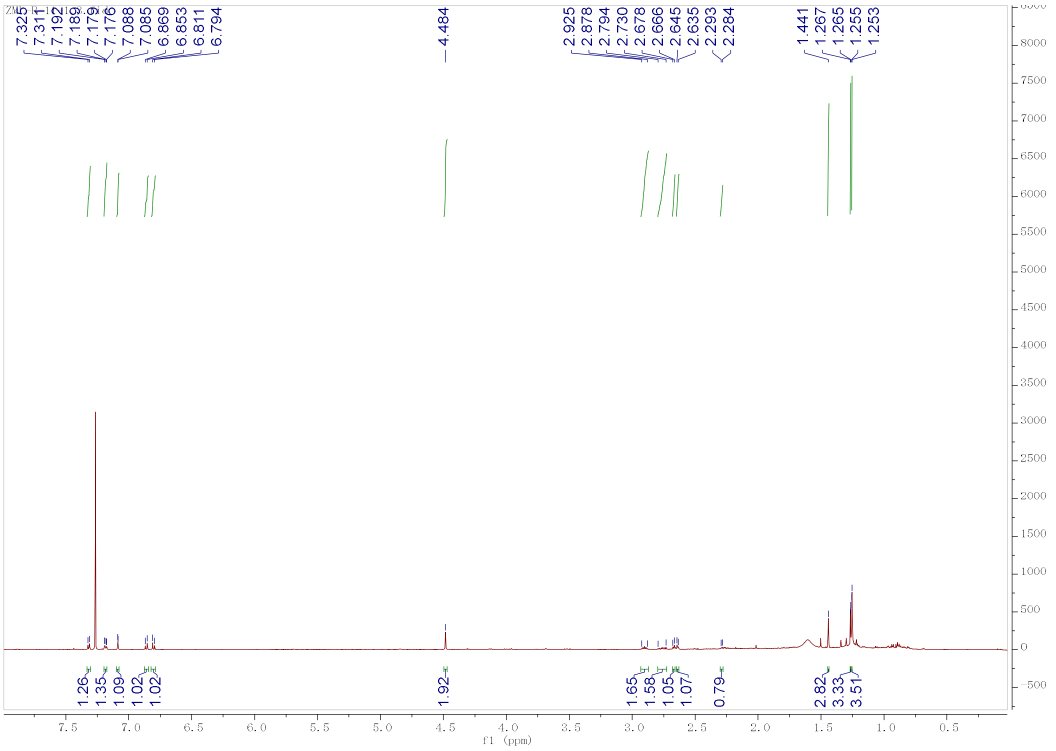

Figure S1.1 ^{1}H NMR spectrum of compound **1** (600 MHz, $CDCl_3$)

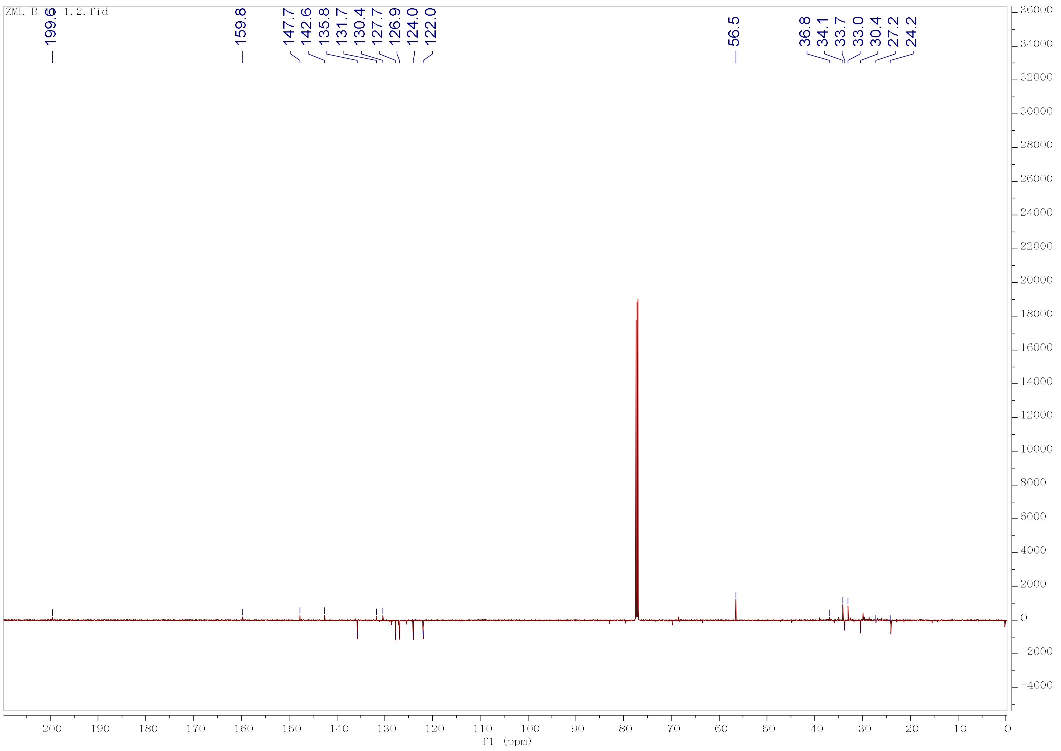

Figure S1.2 ^{13}C APT spectrum of compound **1** (150 MHz, $CDCl_3$)

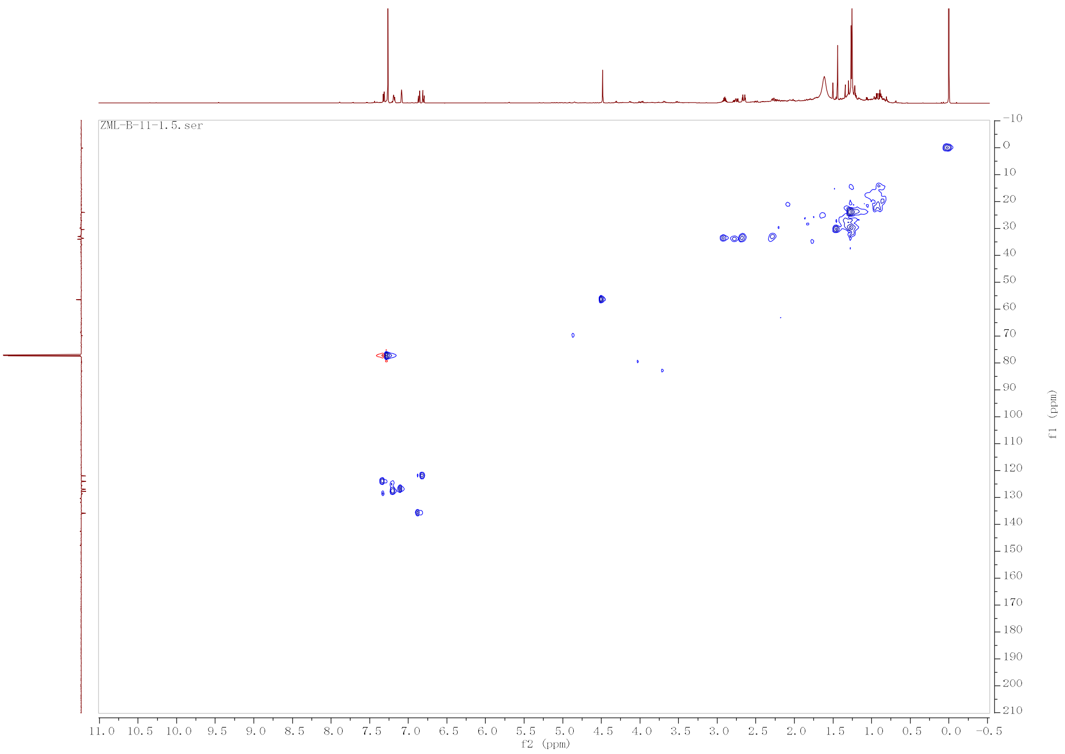

Figure S1.3 HSQC spectrum of compound **1**

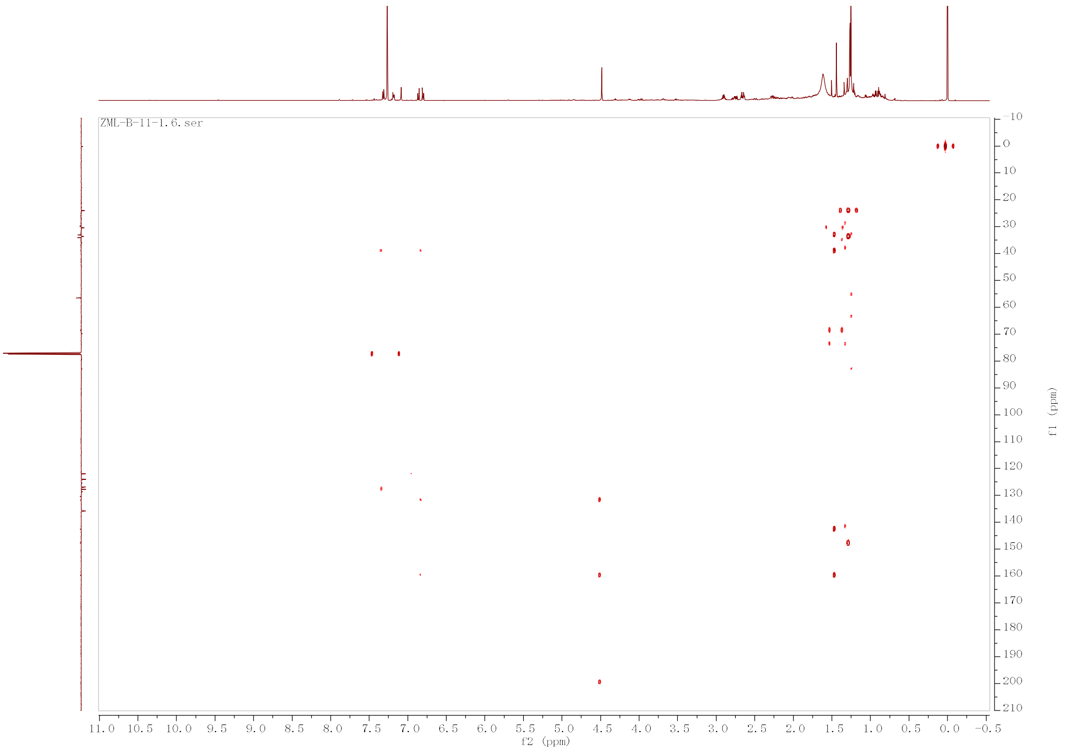

Figure S1.4 HMBC spectrum of compound **1**

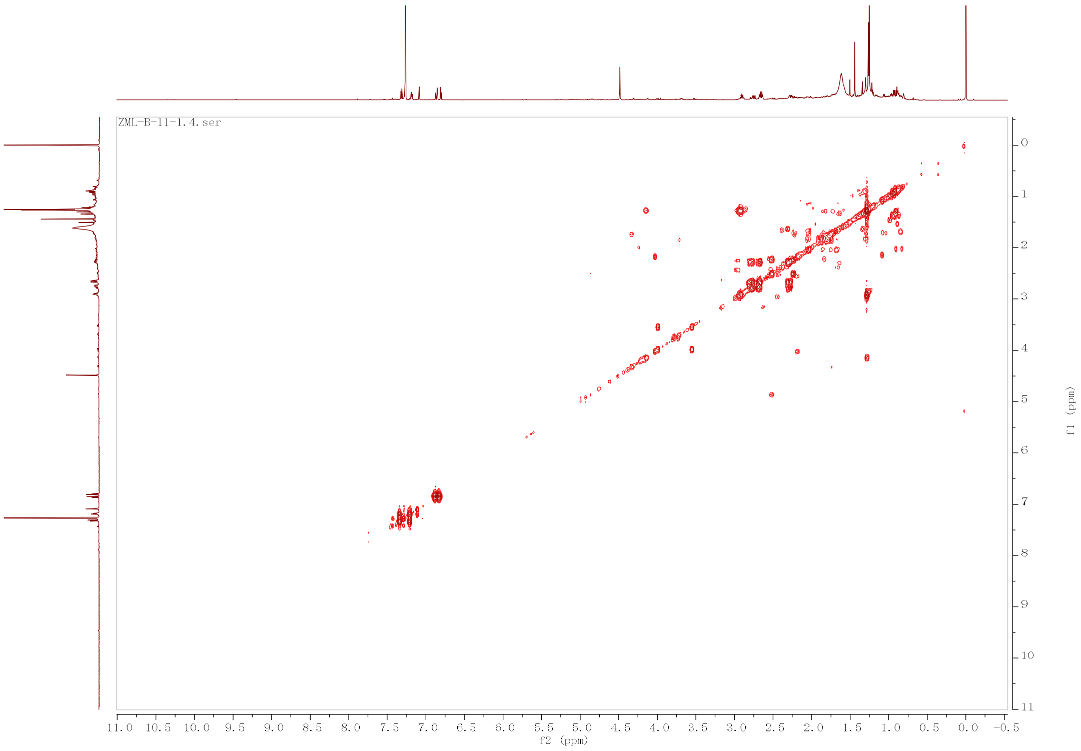

Figure S1.5 ¹H-¹H COSY spectrum of compound **1**

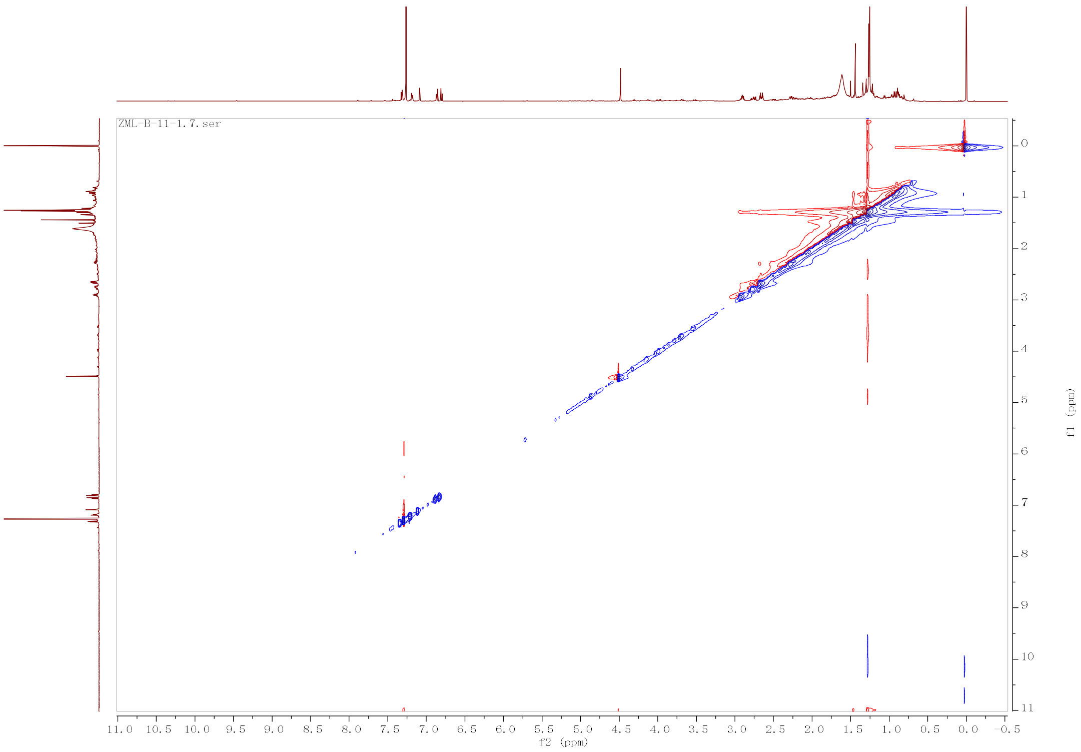

Figure S1.6 NOESY spectrum of compound **1**

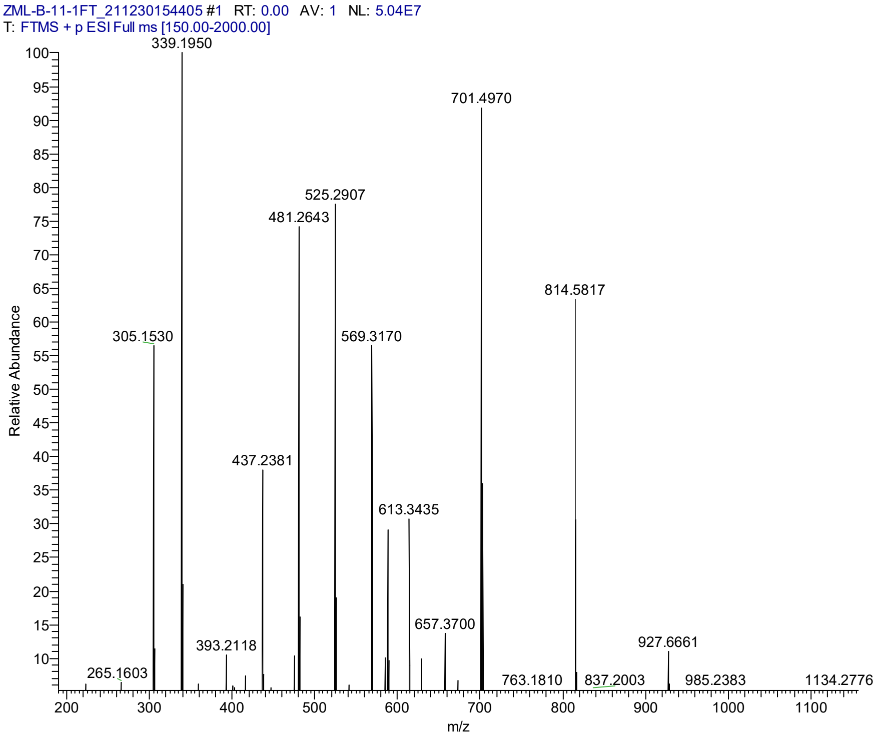

Figure S1.7 Positive ion HRESIMS of compound **1**

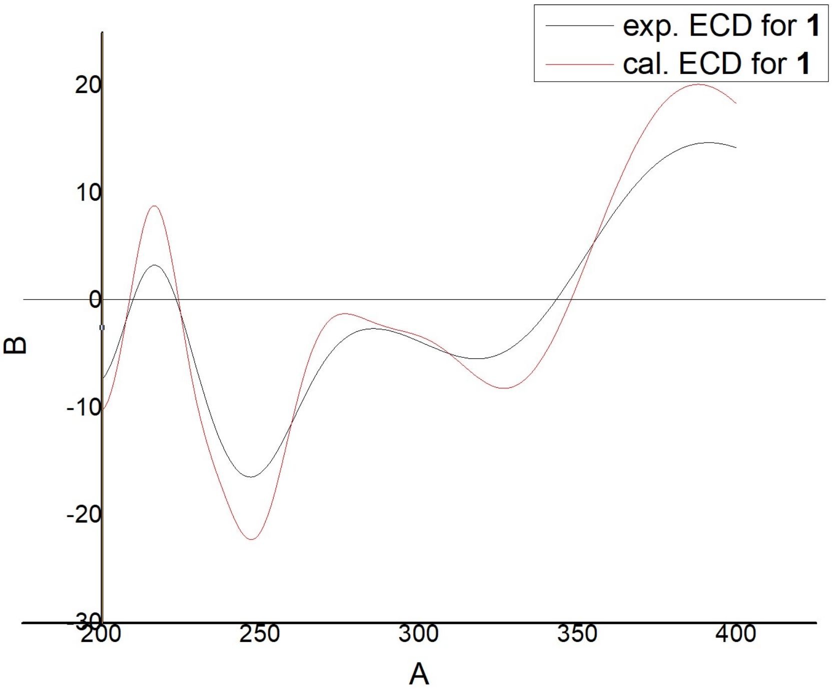

Figure S1.8 Experimental and calculated ECD spectra of compound **1**

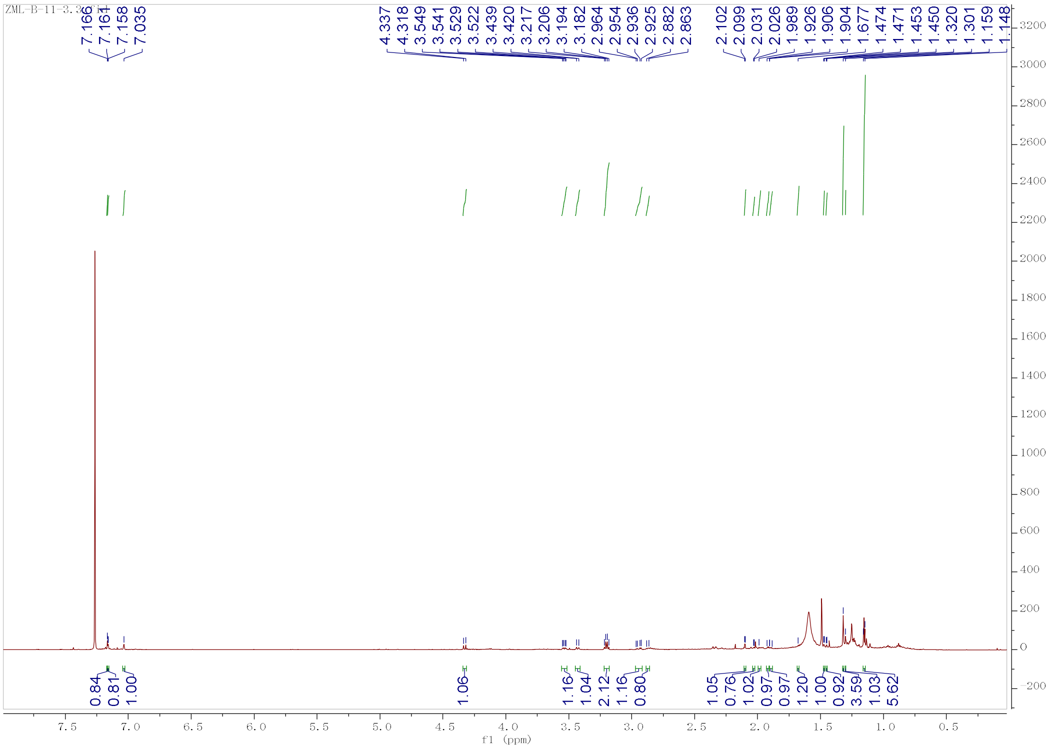

Figure S2.1 ^{1}H NMR spectrum of compound **2** (600 MHz, $CDCl_3$)

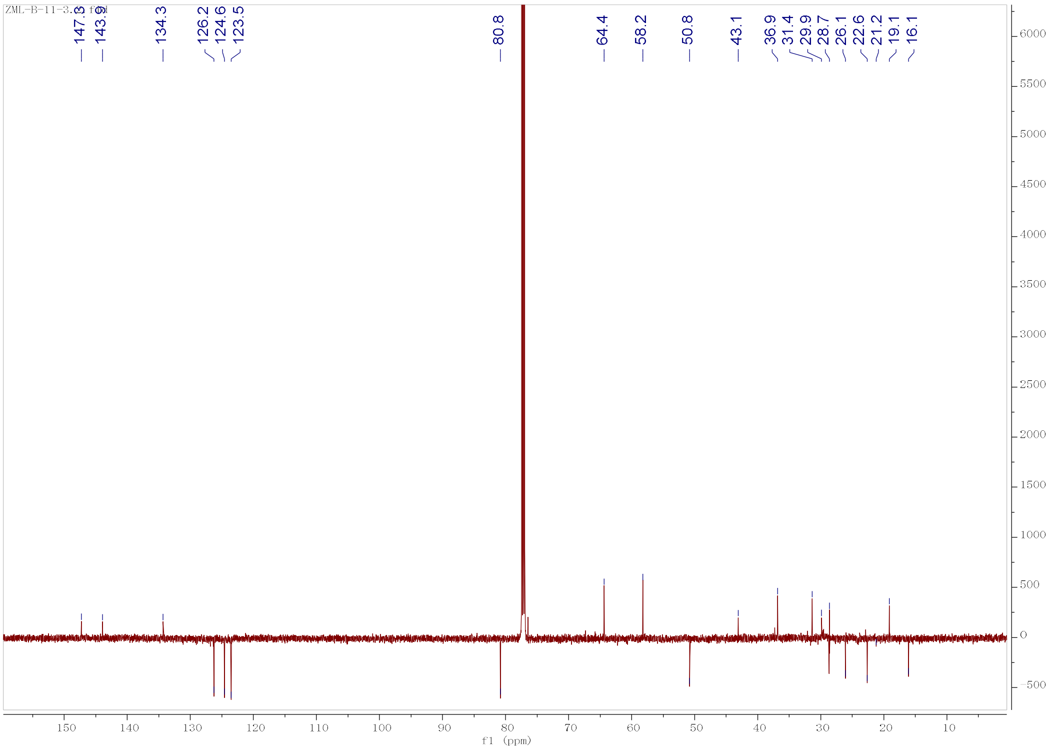

Figure S2.2 ^{13}C APT spectrum of compound **2** (150 MHz, $CDCl_3$)

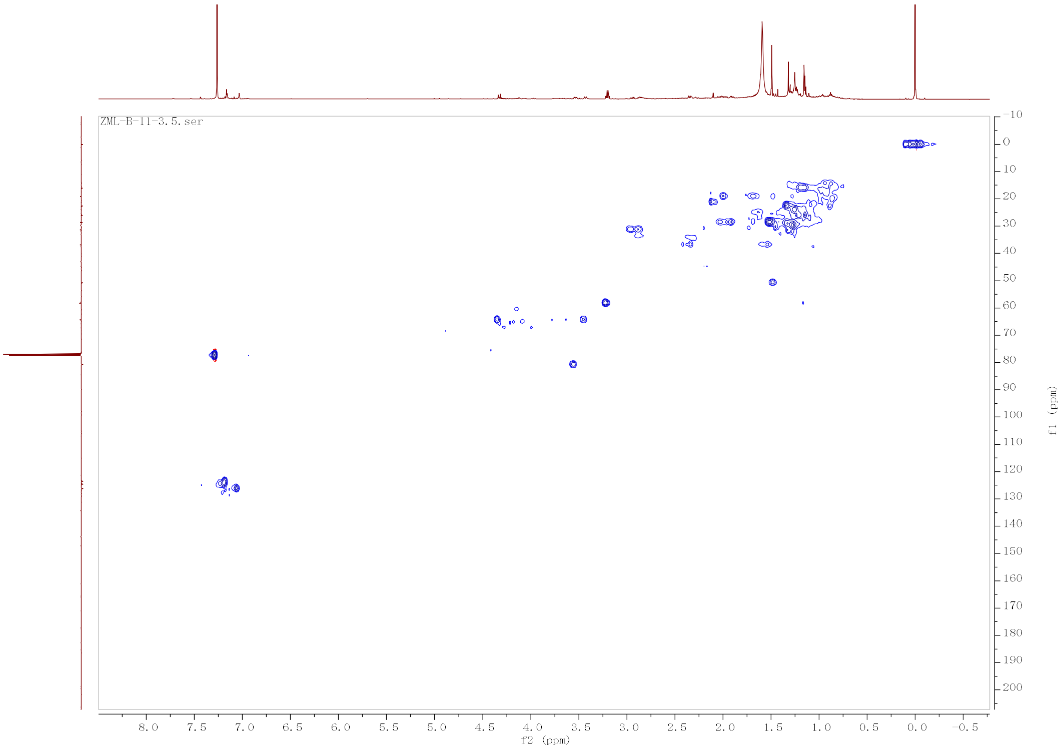

Figure S2.3 HSQC spectrum of compound **2**

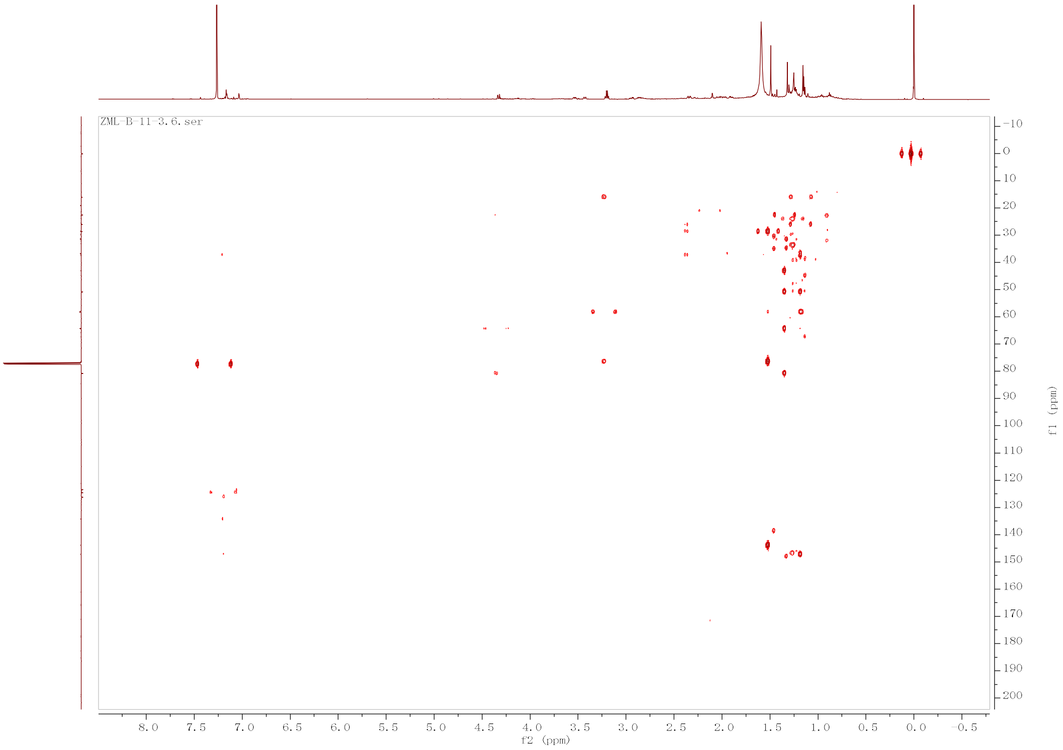

Figure S2.4 HMBC spectrum of compound **2**

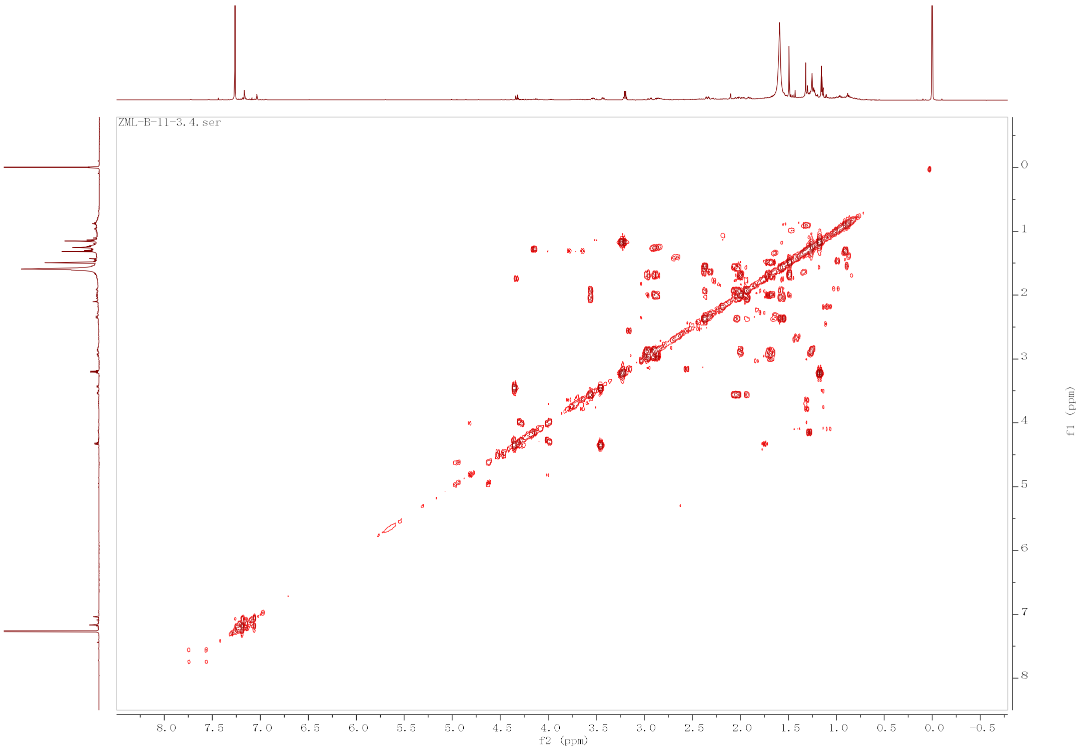

Figure S2.5 ^1H-^1H COSY spectrum of compound **2**

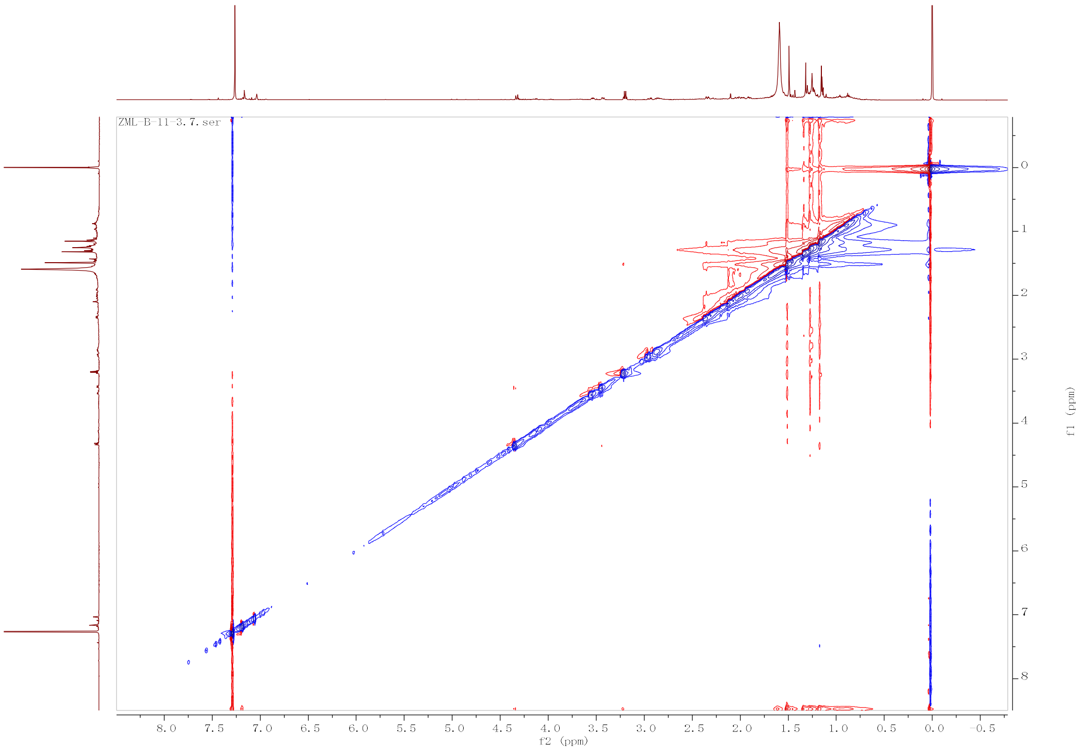

Figure S2.6 NOESY spectrum of compound **2**

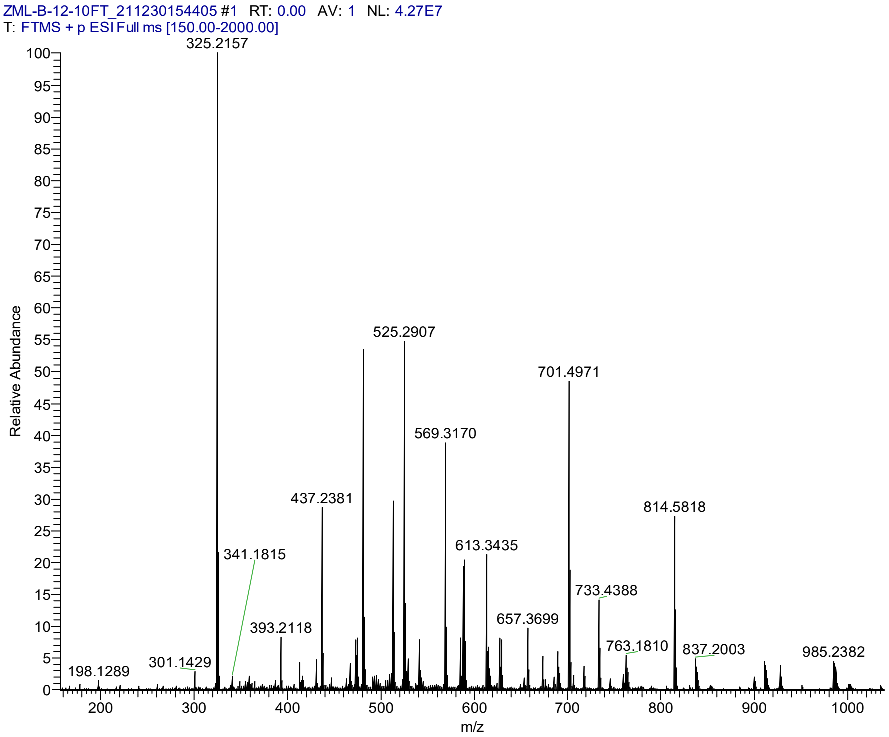

Figure S2.7 Positive ion HRESIMS of compound **2**

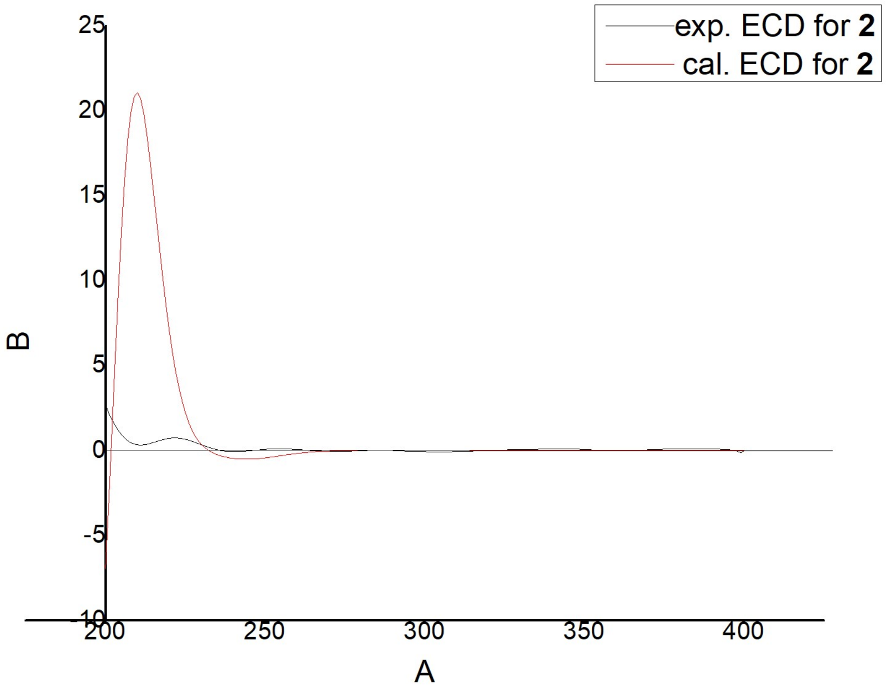

Figure S2.8 Experimental and calculated ECD spectra of compound **2**

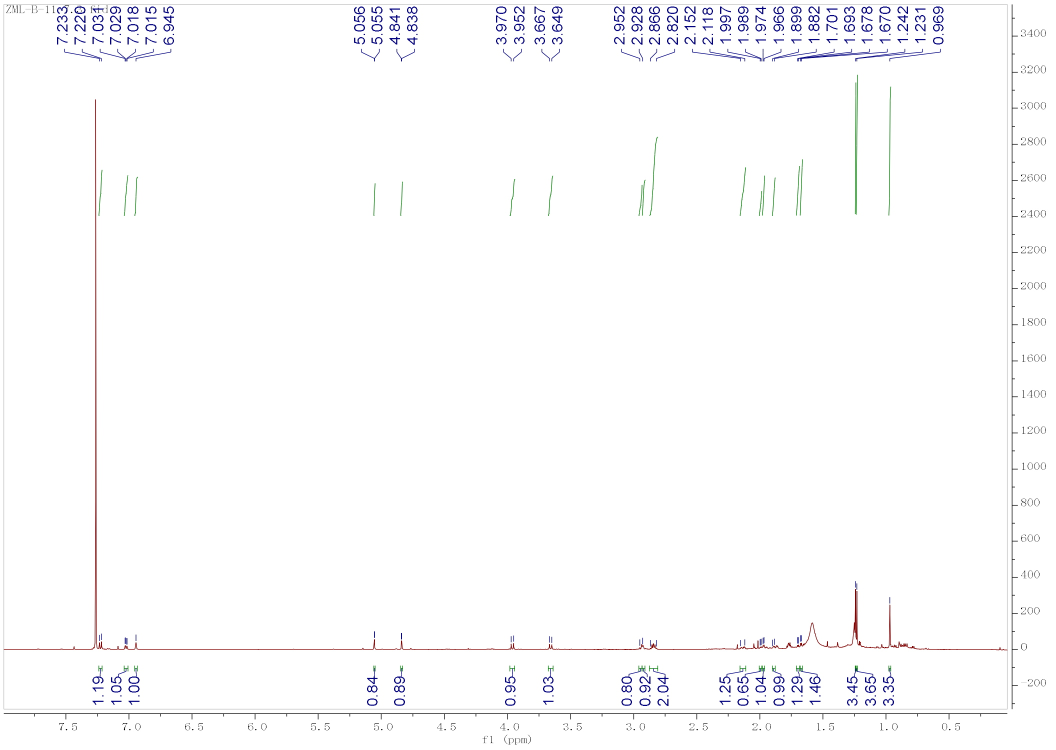

Figure S3.1 ^{1}H NMR spectrum of compound **3** (600 MHz, $CDCl_3$)

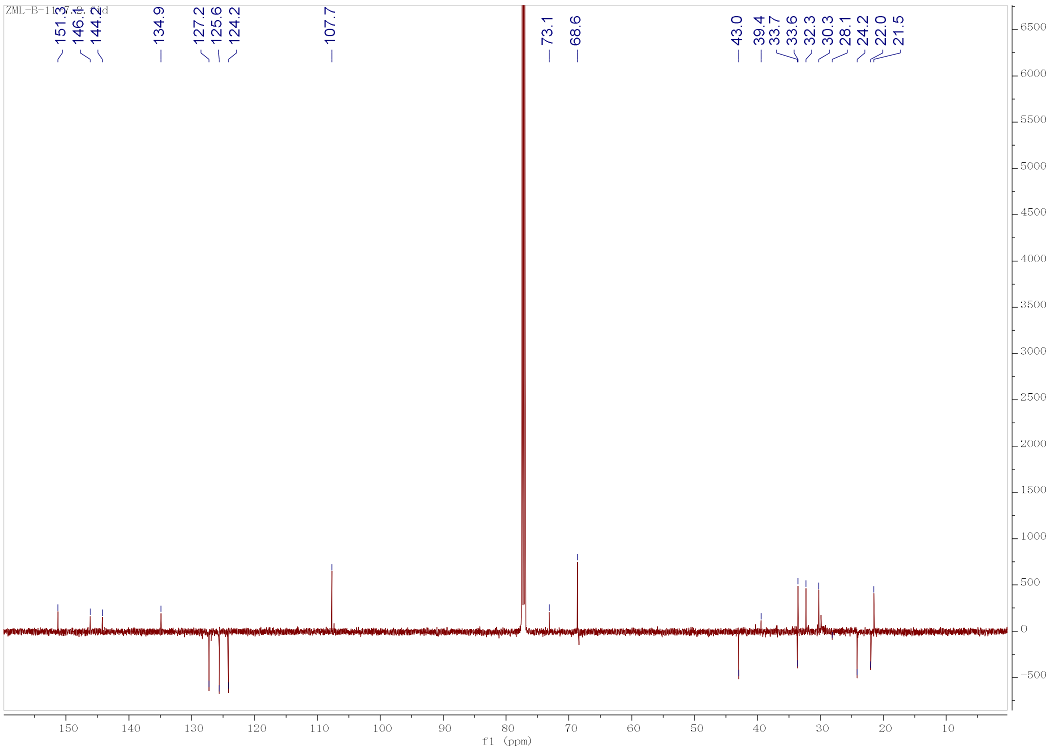

Figure S3.2 ^{13}C APT spectrum of compound **3** (150 MHz, $CDCl_3$)

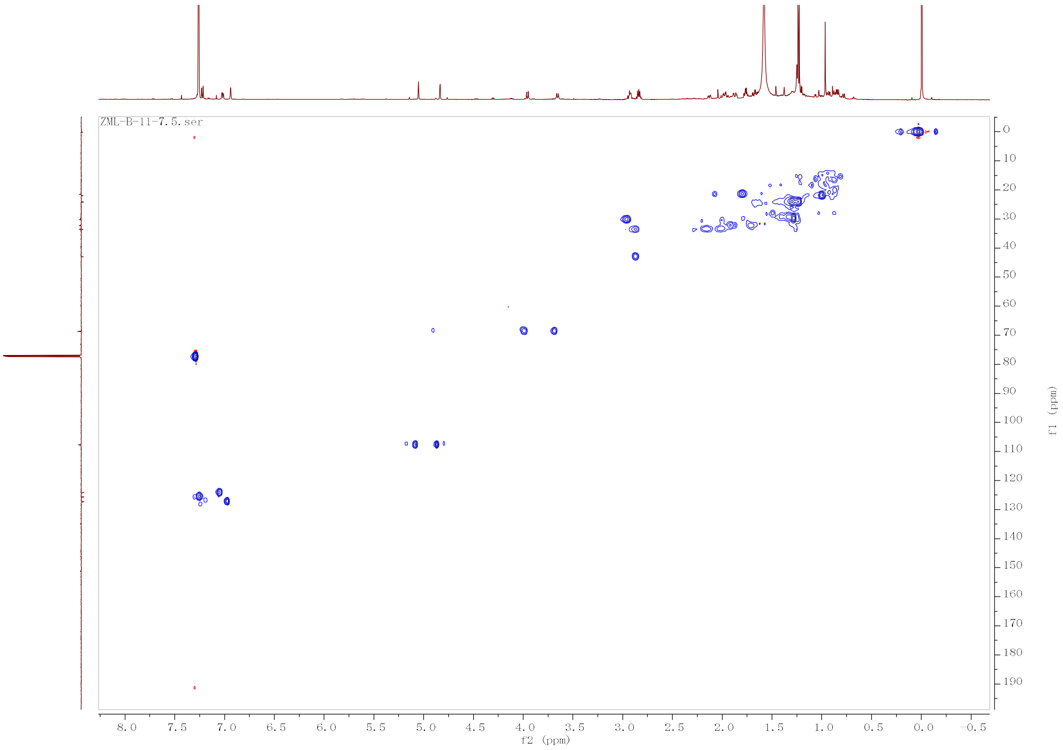

Figure S3.3 HSQC spectrum of compound **3**

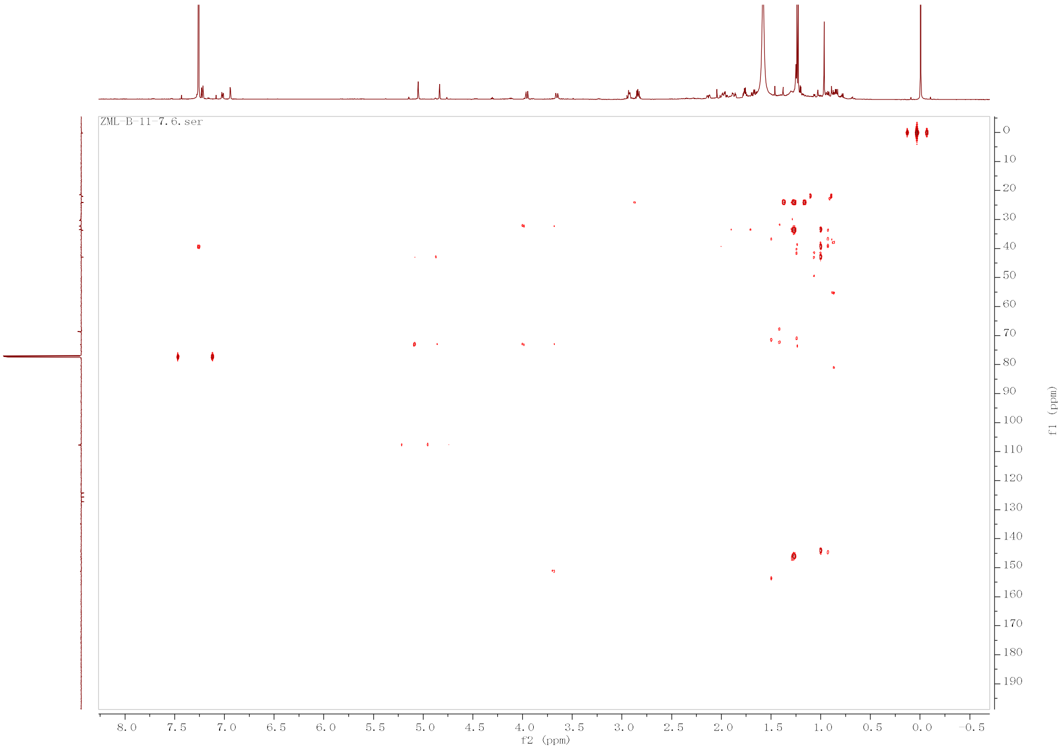

Figure S3.4 HMBC spectrum of compound **3**

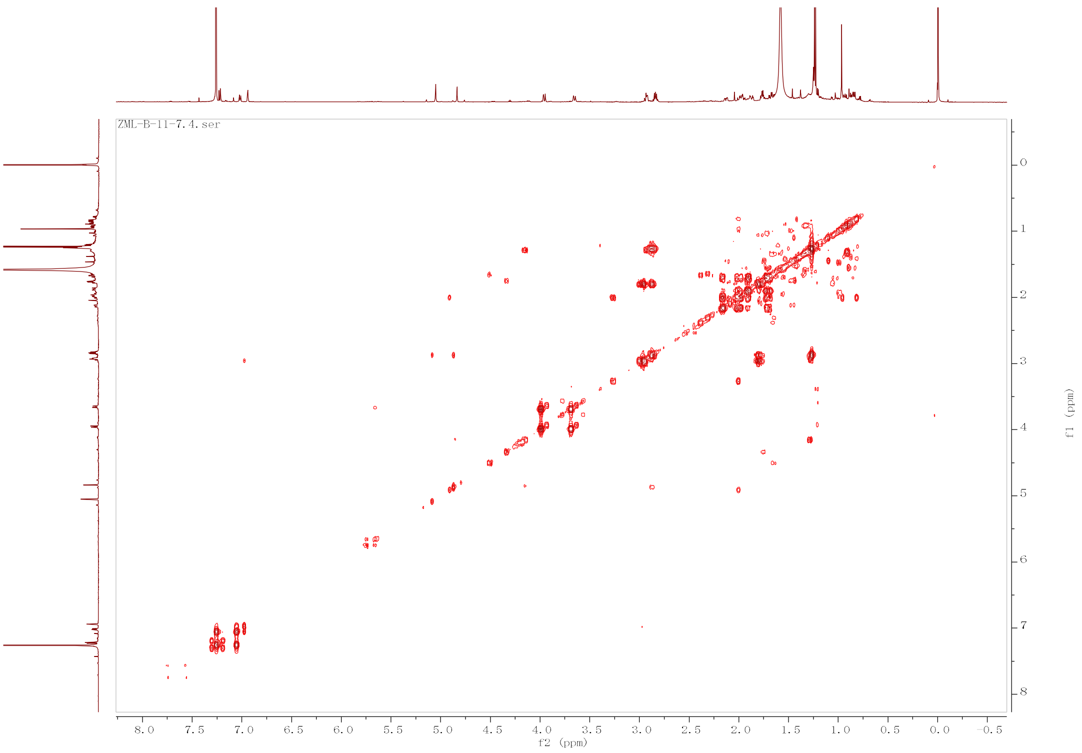

Figure S3.5 ^{1}H-^{1}H COSY spectrum of compound **3**

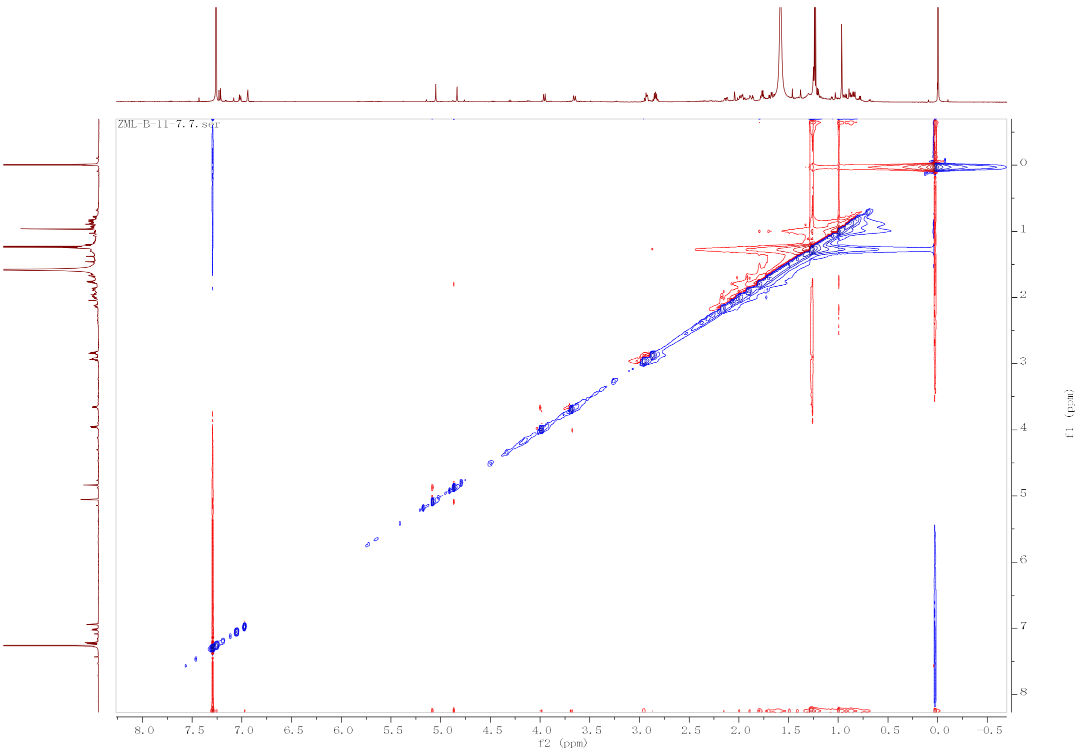

Figure S3.6 NOESY spectrum of compound **3**

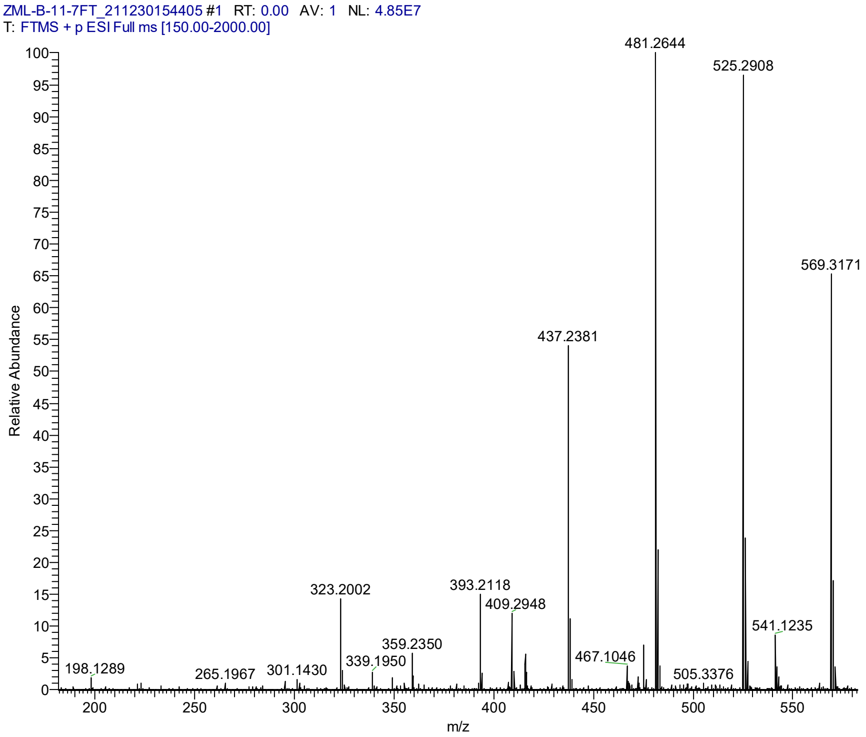

Figure S3.7 Positive ion HRESIMS of compound **3**

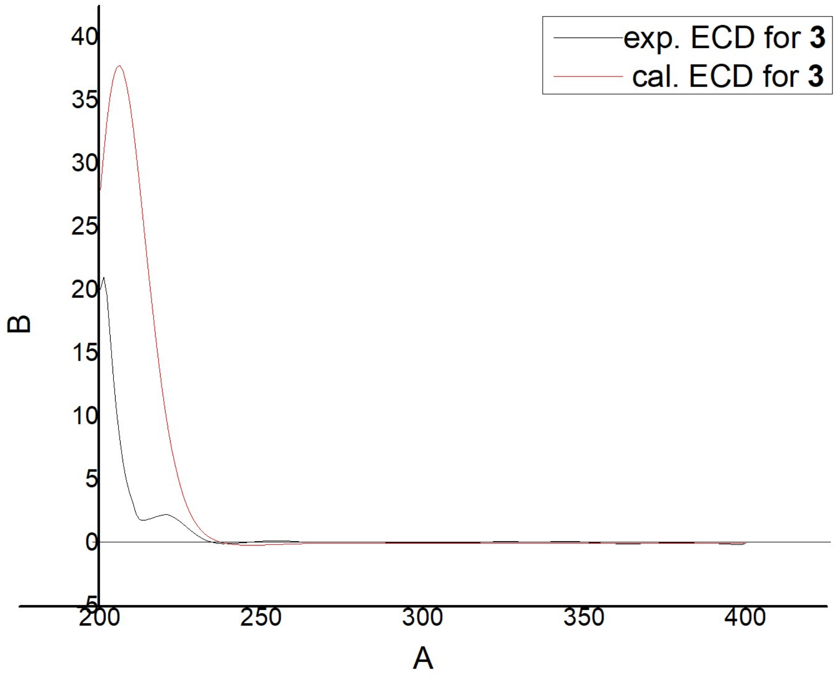

Figure S3.8 Experimental and calculated ECD spectra of compound **3**

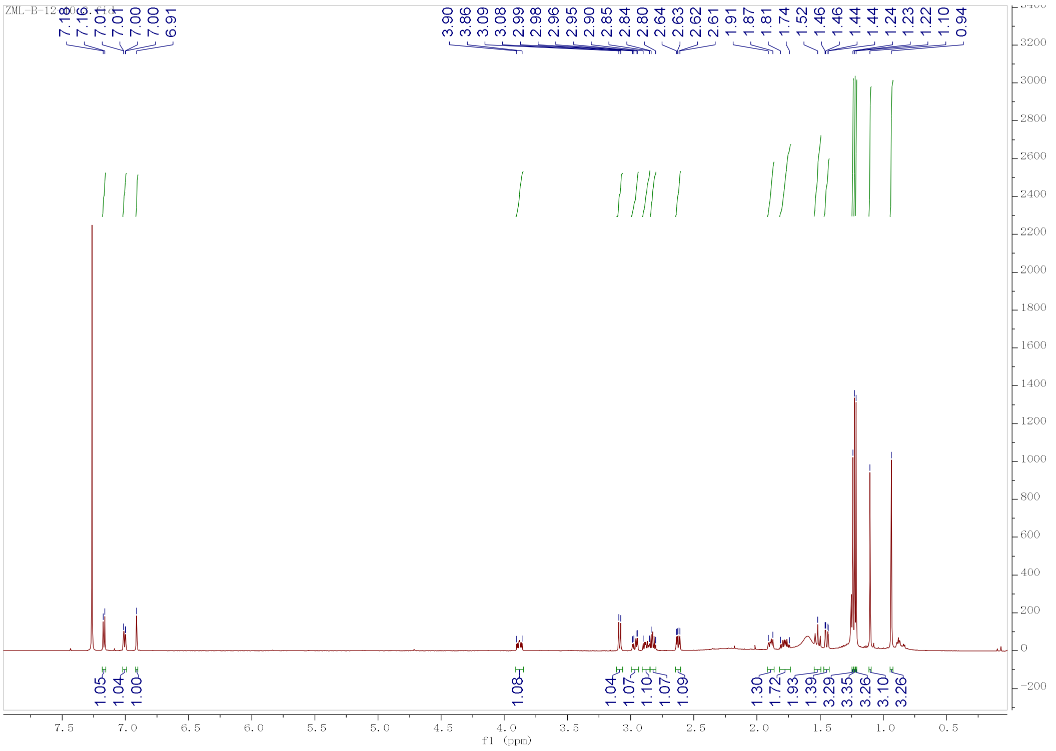

Figure S4.1 ^{1}H NMR spectrum of compound **4** (600 MHz, $CDCl_3$)

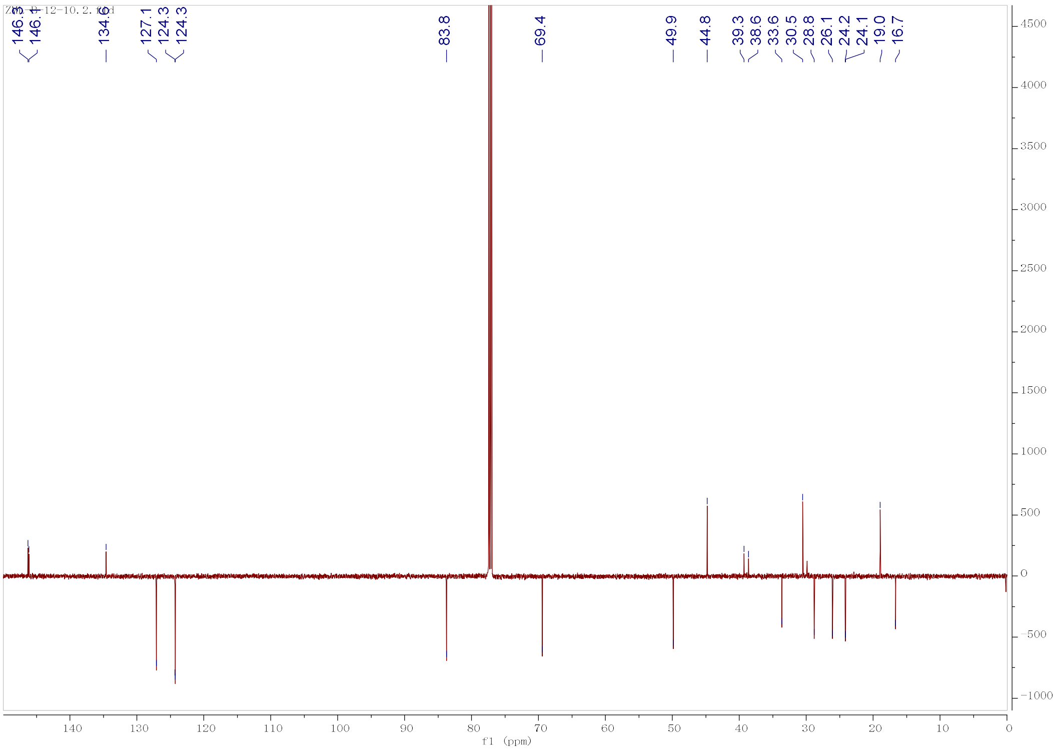

Figure S4.2 ^{13}C APT spectrum of compound **4** (150 MHz, $CDCl_3$)

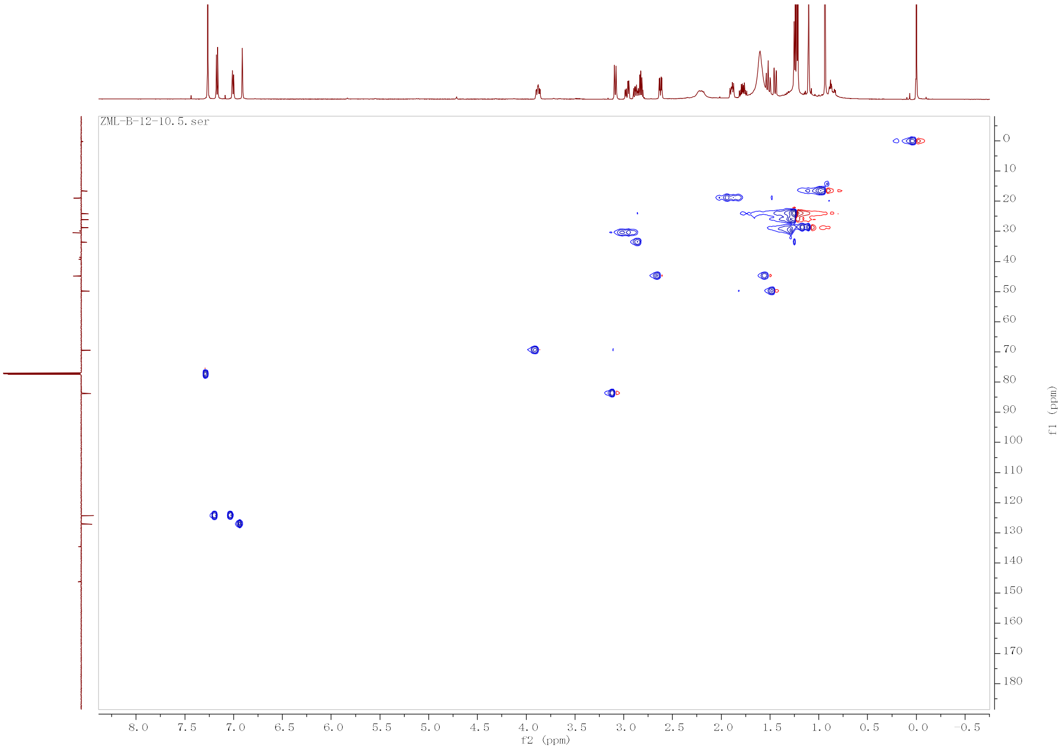

Figure S4.3 HSQC spectrum of compound **4**

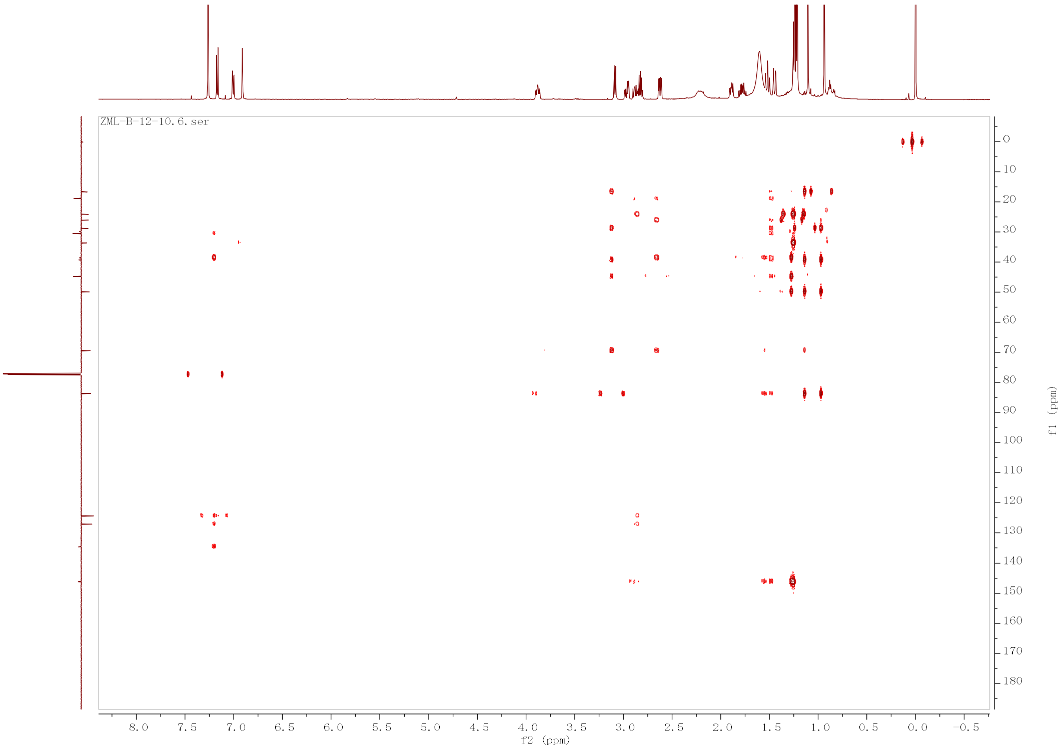

Figure S4.4 HMBC spectrum of compound **4**

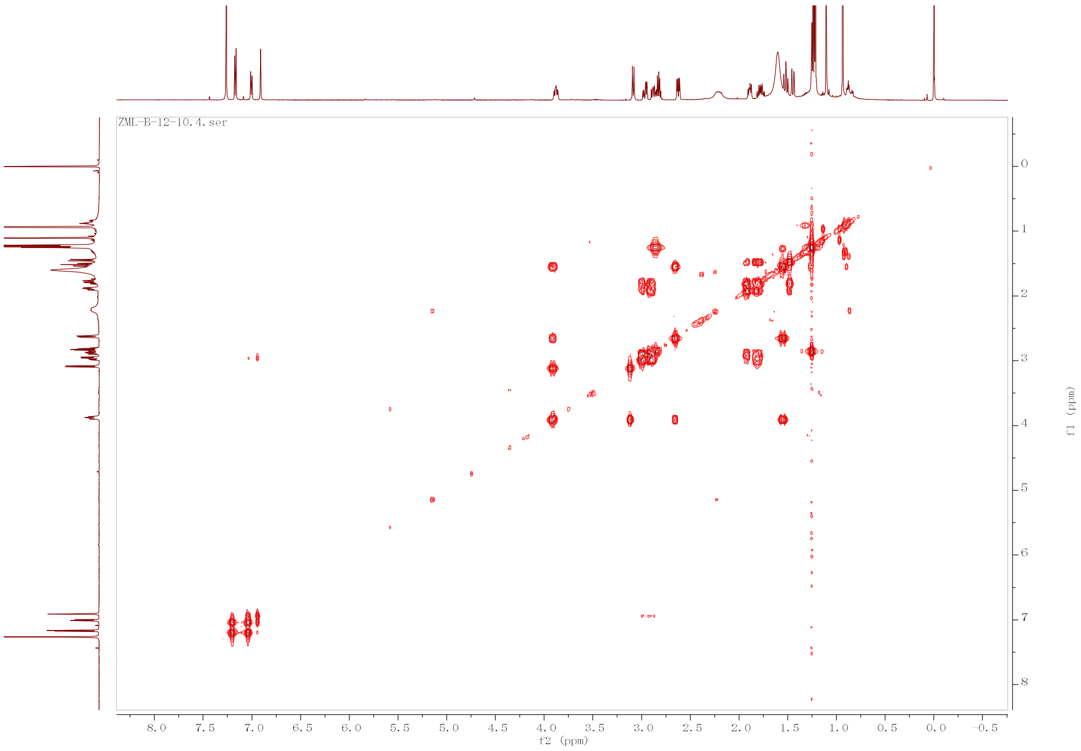

Figure S4.5 ^1H-^1H COSY spectrum of compound **4**

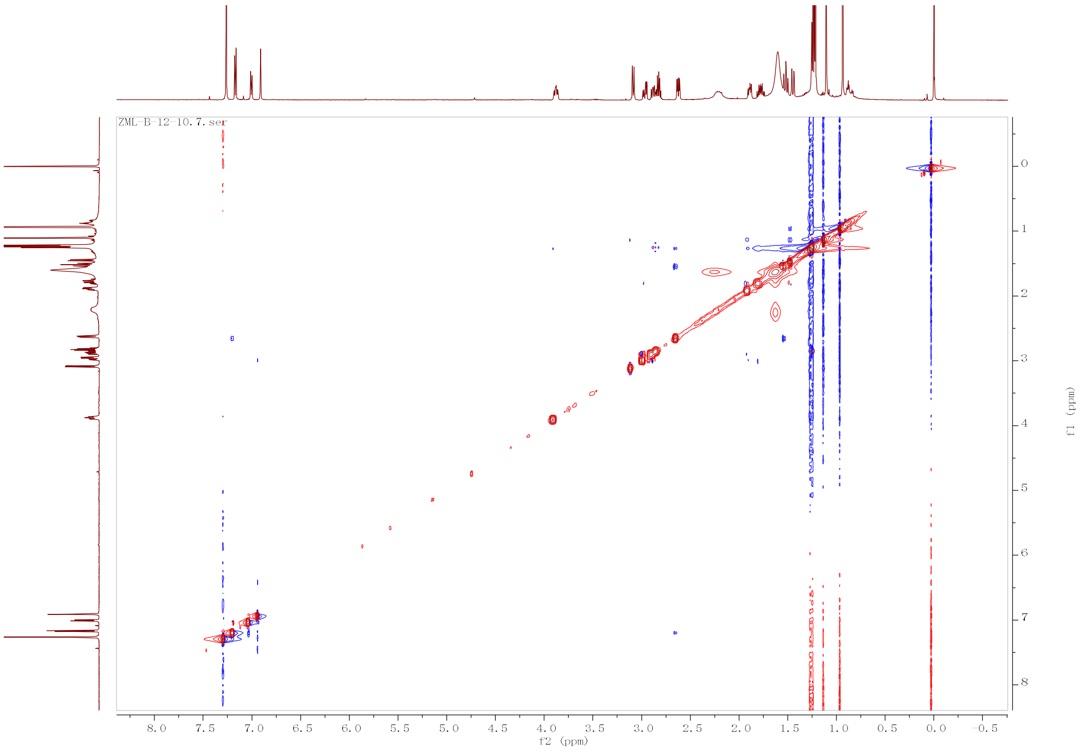

Figure S4.6 NOESY spectrum of compound **4**

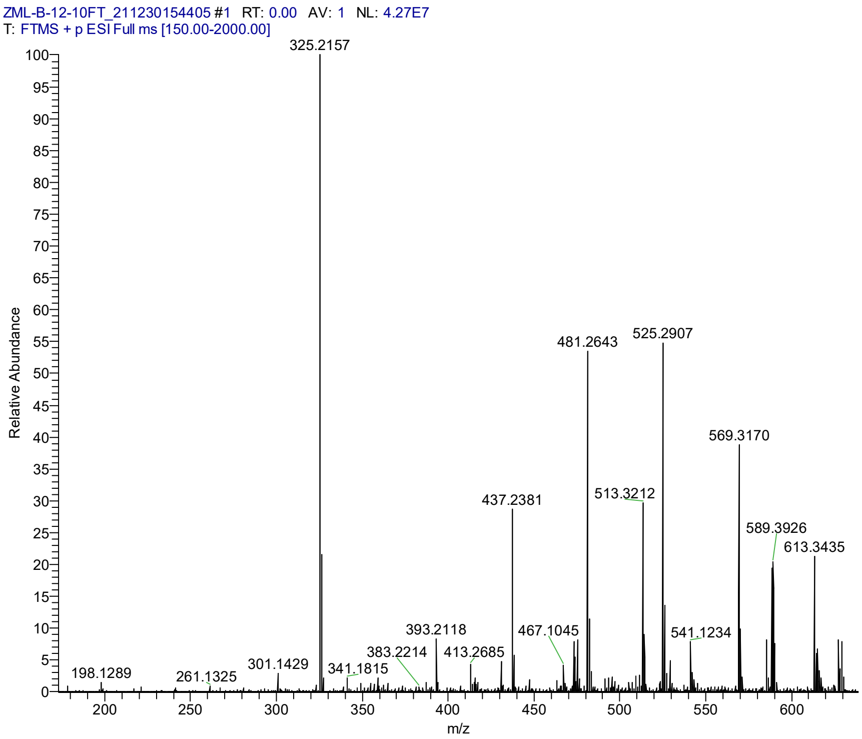

Figure S4.7 Positive ion HRESIMS of compound **4**

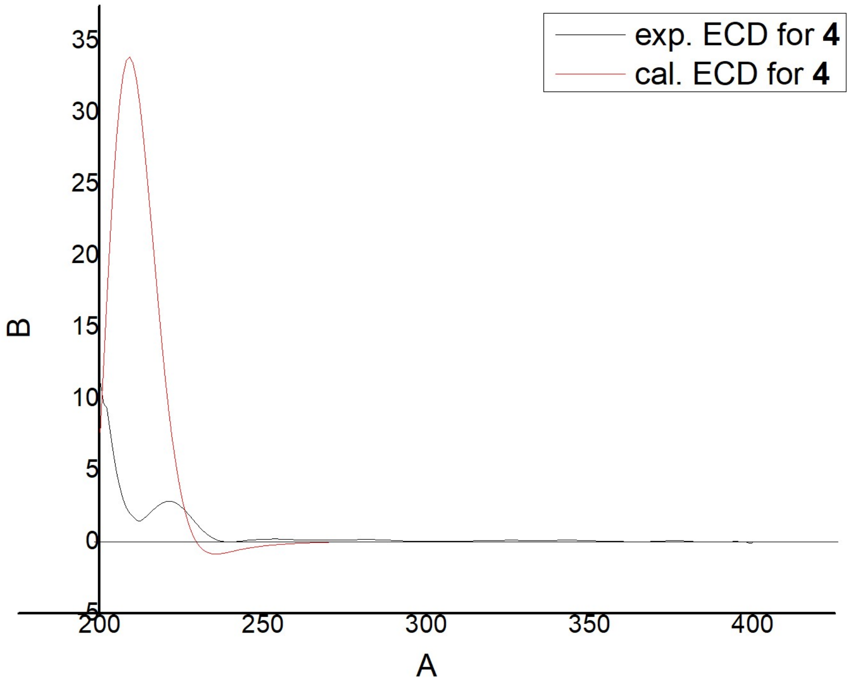

Figure S4.8 Experimental and calculated ECD spectra of compound **4**

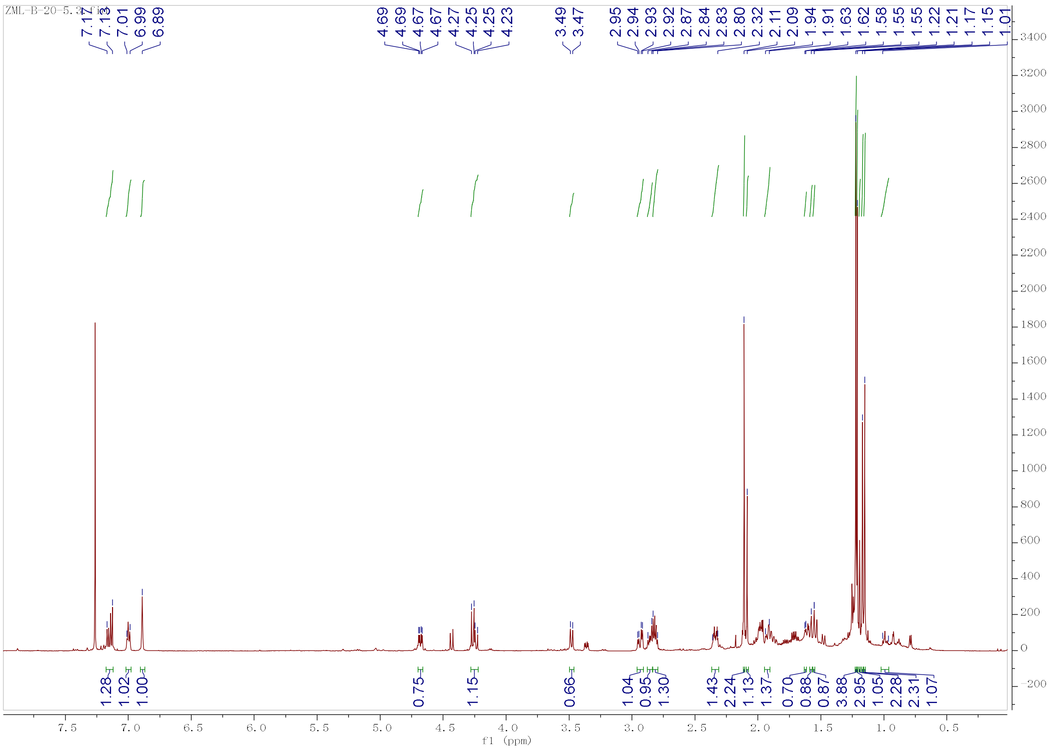

Figure S5.1 ^{1}H NMR spectrum of compound **5** (600 MHz, $CDCl_3$)

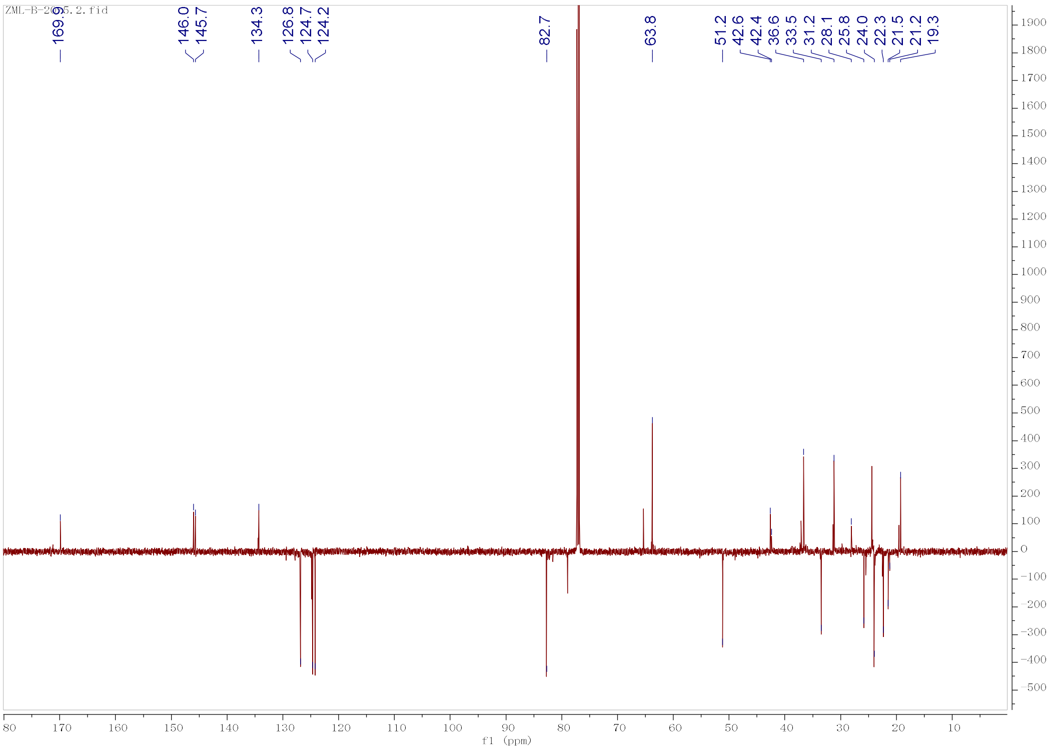

Figure S5.2 ^{13}C APT spectrum of compound **5** (150 MHz, $CDCl_3$)

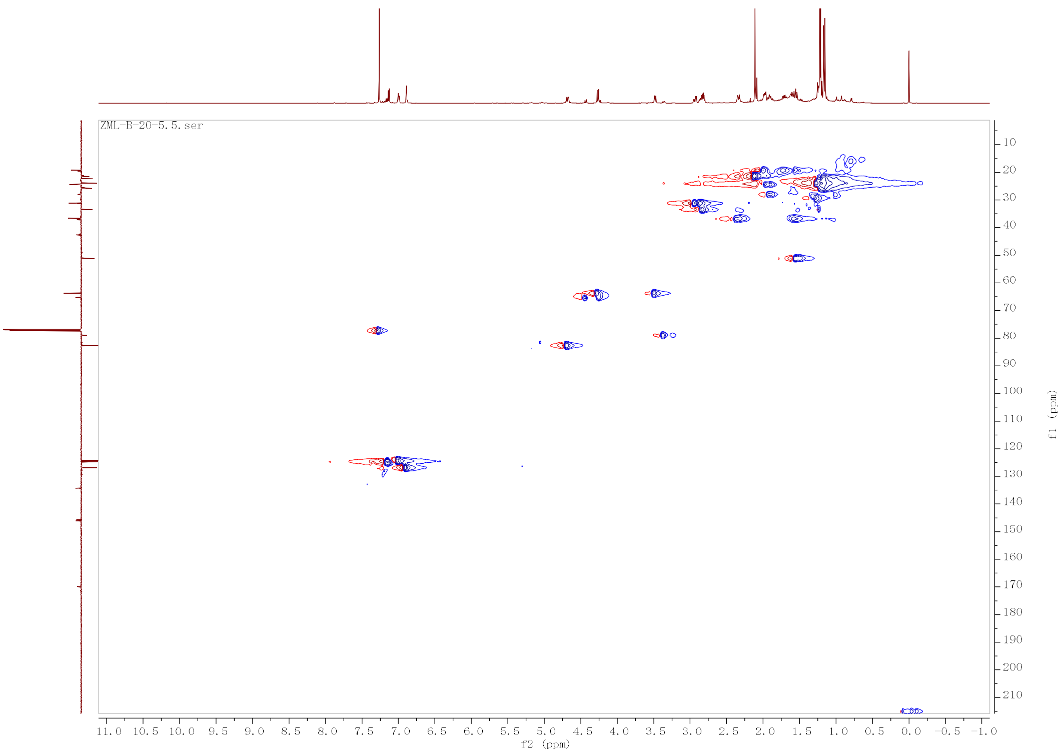

Figure S5.3 HSQC spectrum of compound **5**

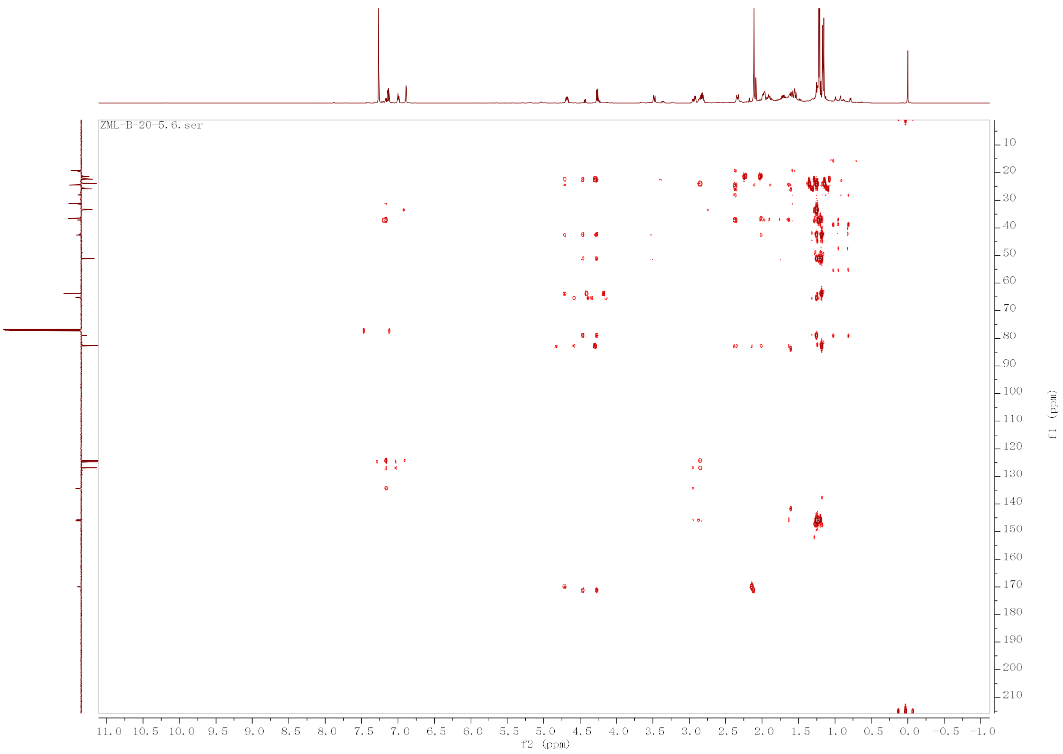

Figure S5.4 HMBC spectrum of compound **5**

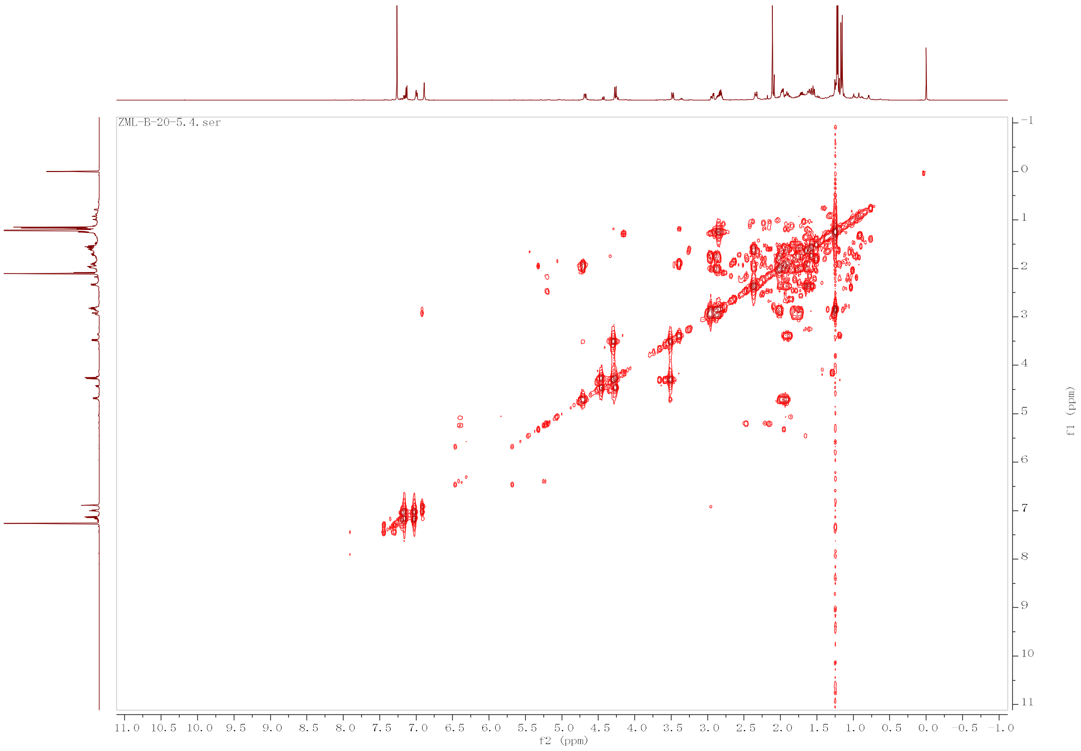

Figure S5.5 ^{1}H-^{1}H COSY spectrum of compound **5**

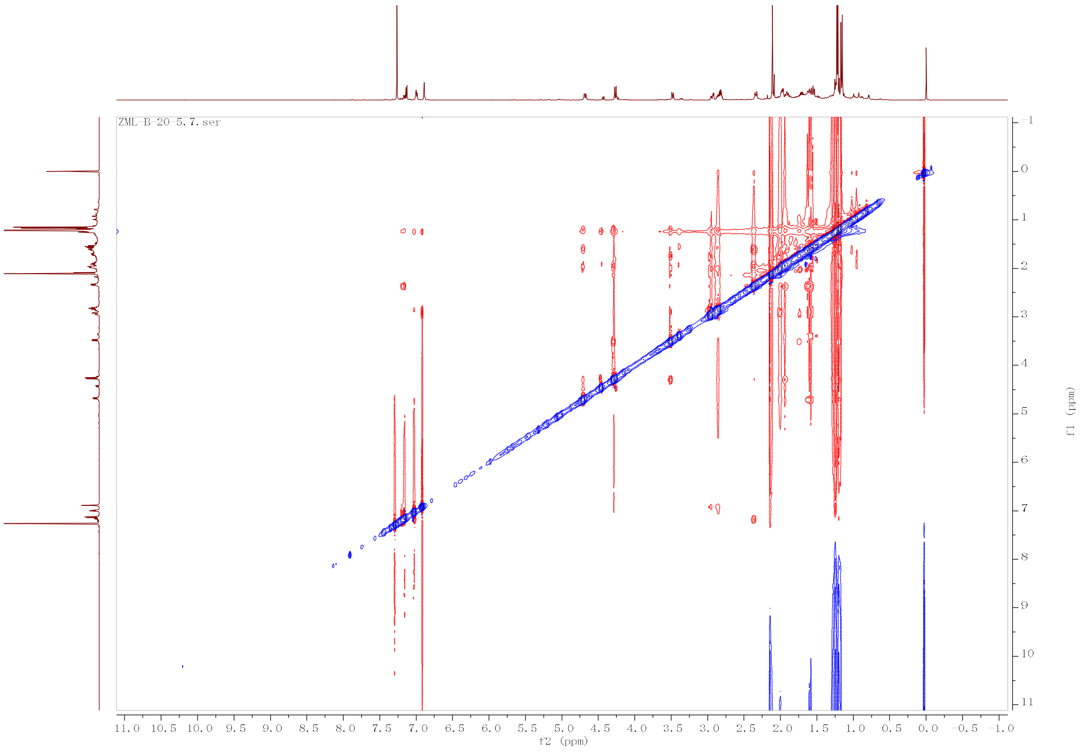

Figure S5.6 NOESY spectrum of compound **5**

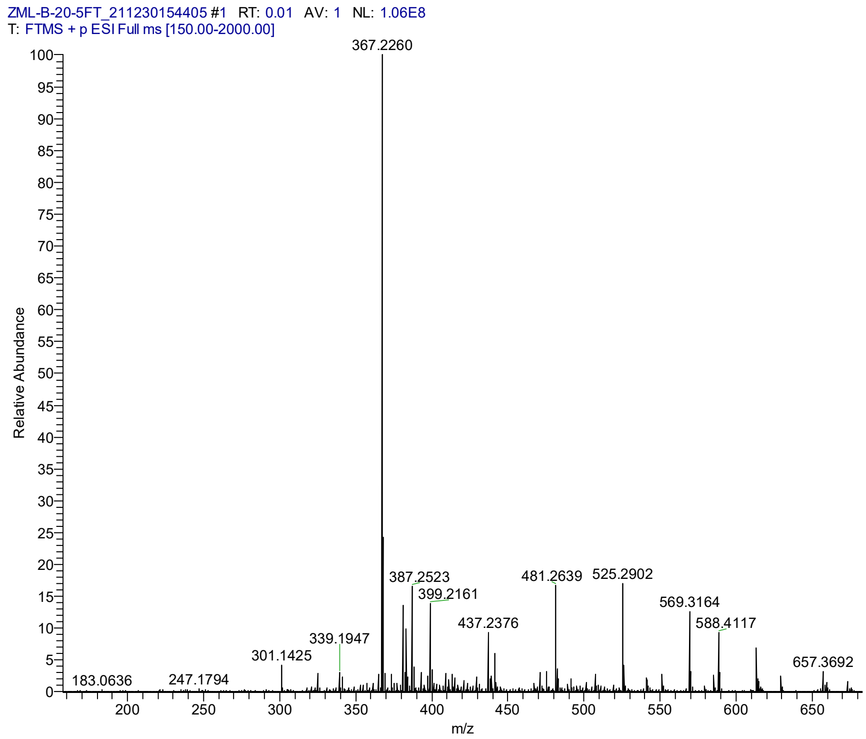

Figure S5.7 Positive ion HRESIMS of compound **5**

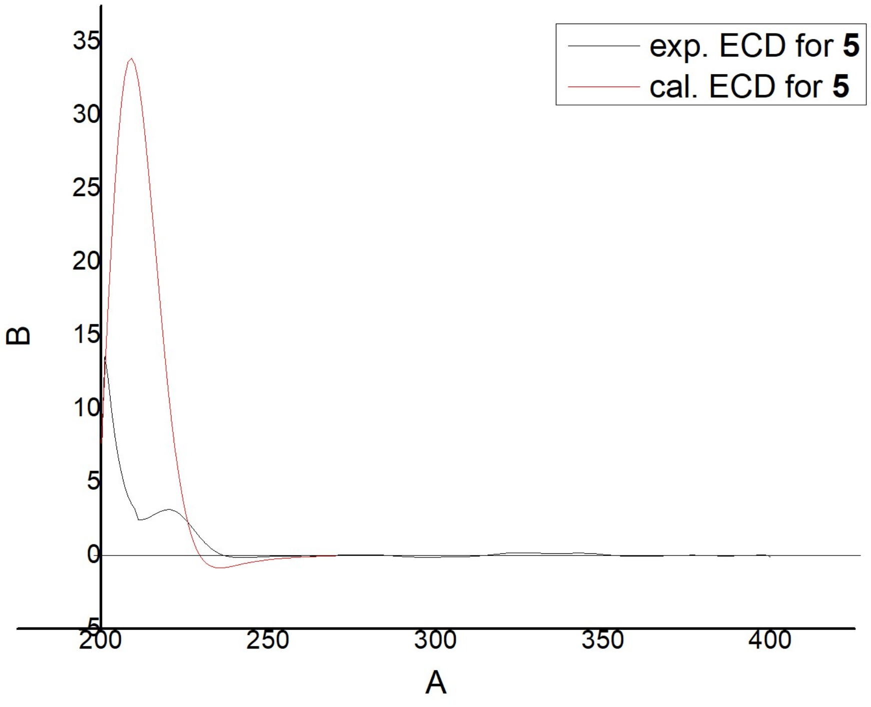

Figure S5.8 Experimental and calculated ECD spectra of compound **5**

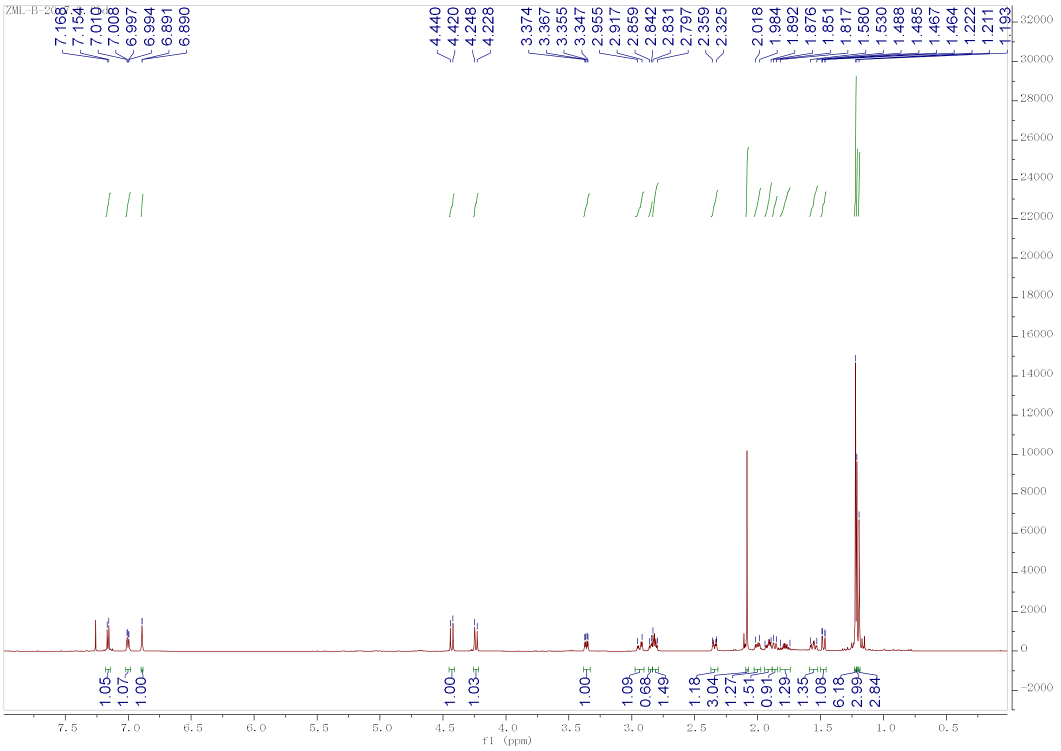

Figure S6.1 ^{1}H NMR spectrum of compound **6** (600 MHz, $CDCl_3$)

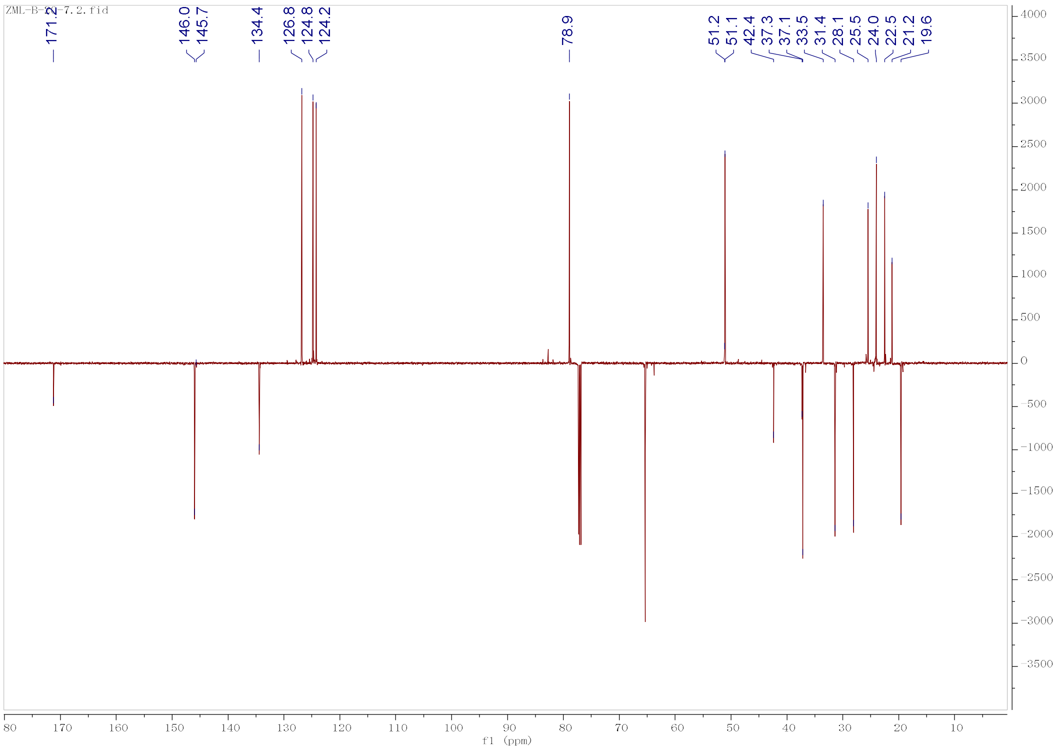

Figure S6.2 ^{13}C APT spectrum of compound **6** (150 MHz, $CDCl_3$)

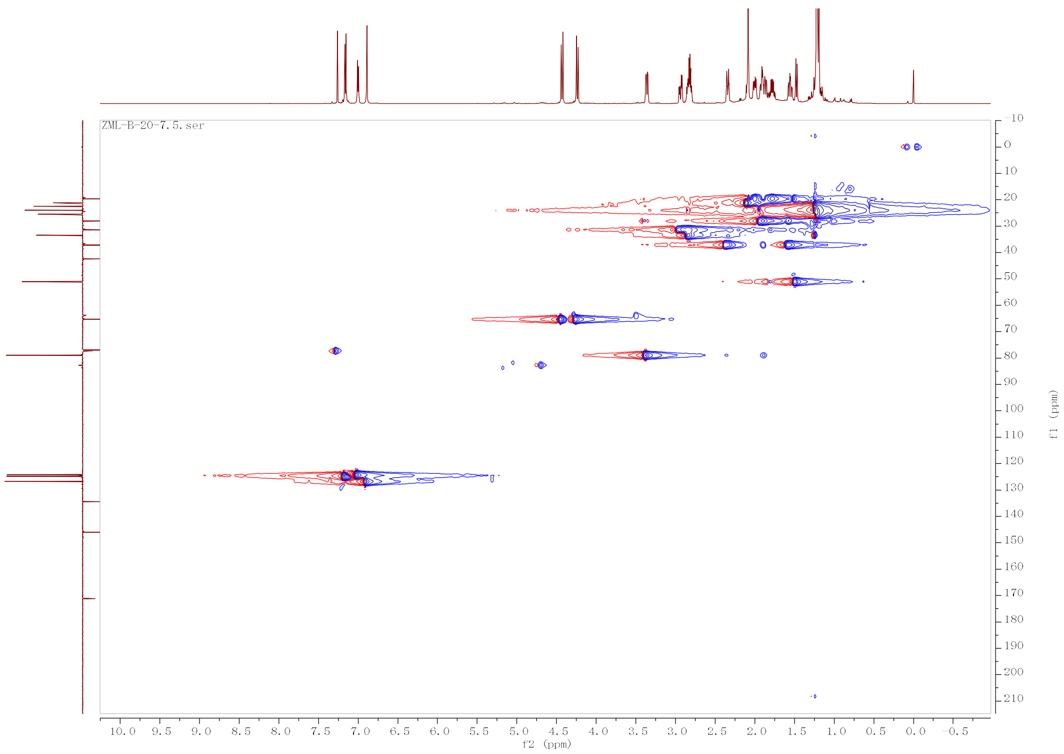

Figure S6.3 HSQC spectrum of compound **6**

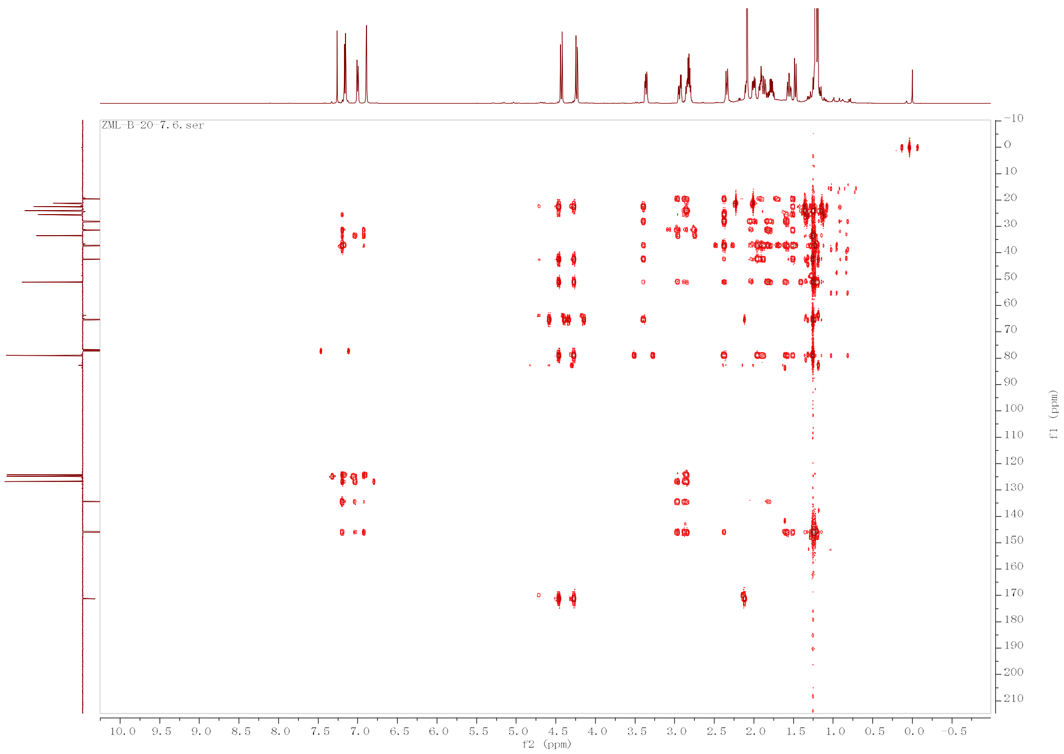

Figure S6.4 HMBC spectrum of compound **6**

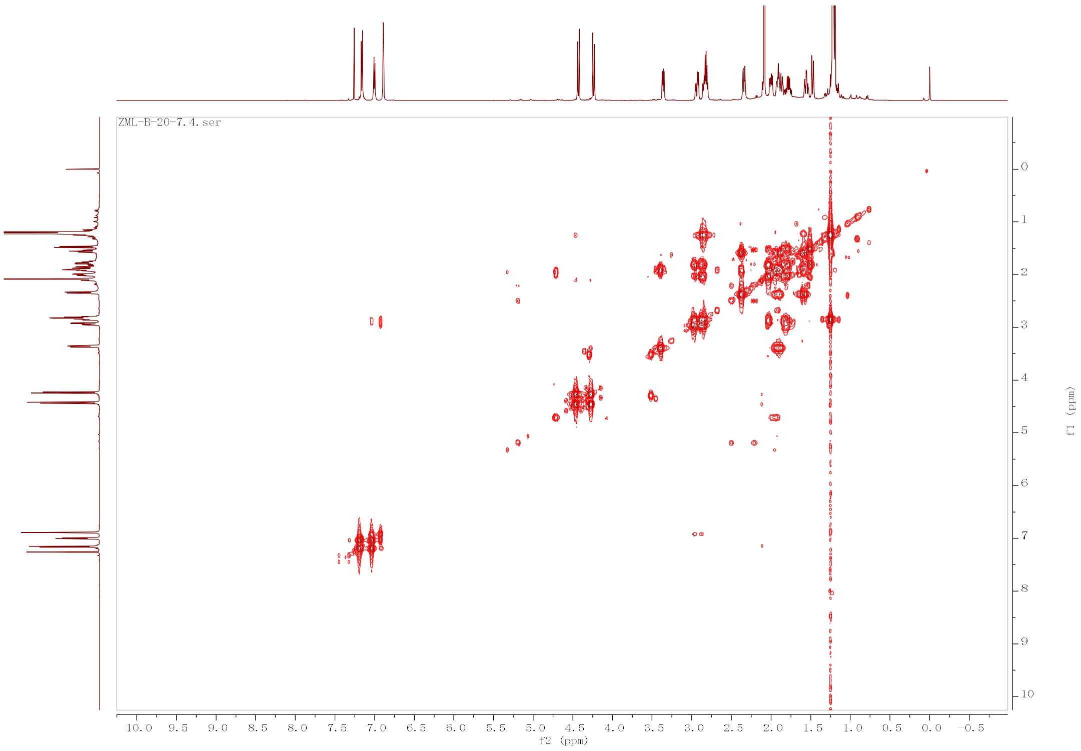

Figure S6.5 ^{1}H-^{1}H COSY spectrum of compound **6**

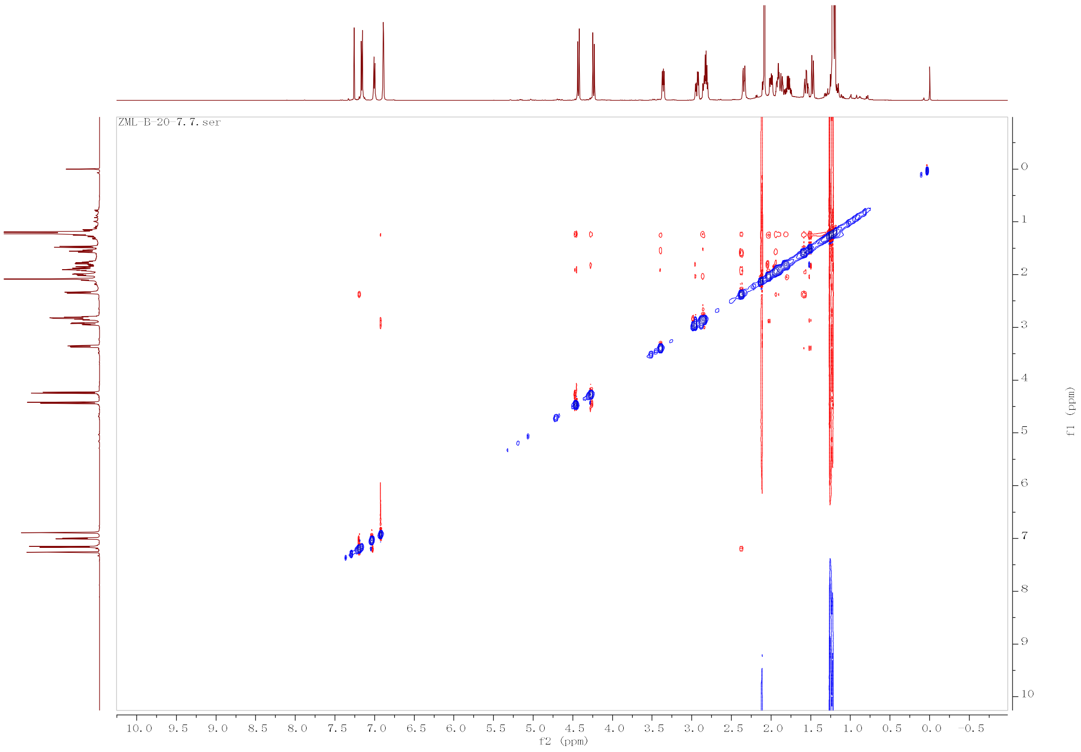

Figure S6.6 NOESY spectrum of compound **6**

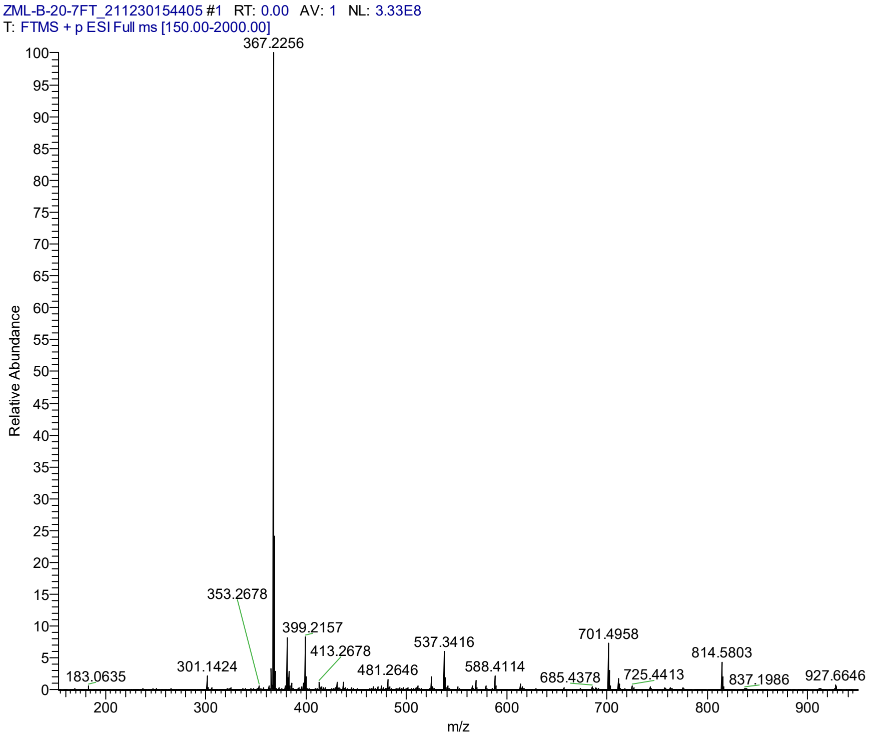

Figure S6.7 Positive ion HRESIMS of compound **6**

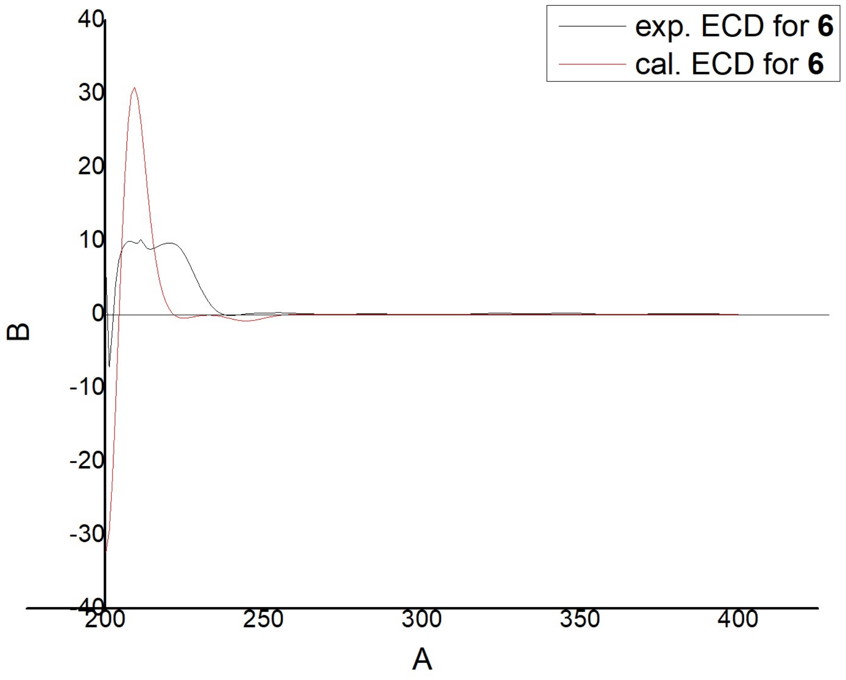

Figure S6.8 Experimental and calculated ECD spectra of compound **6**

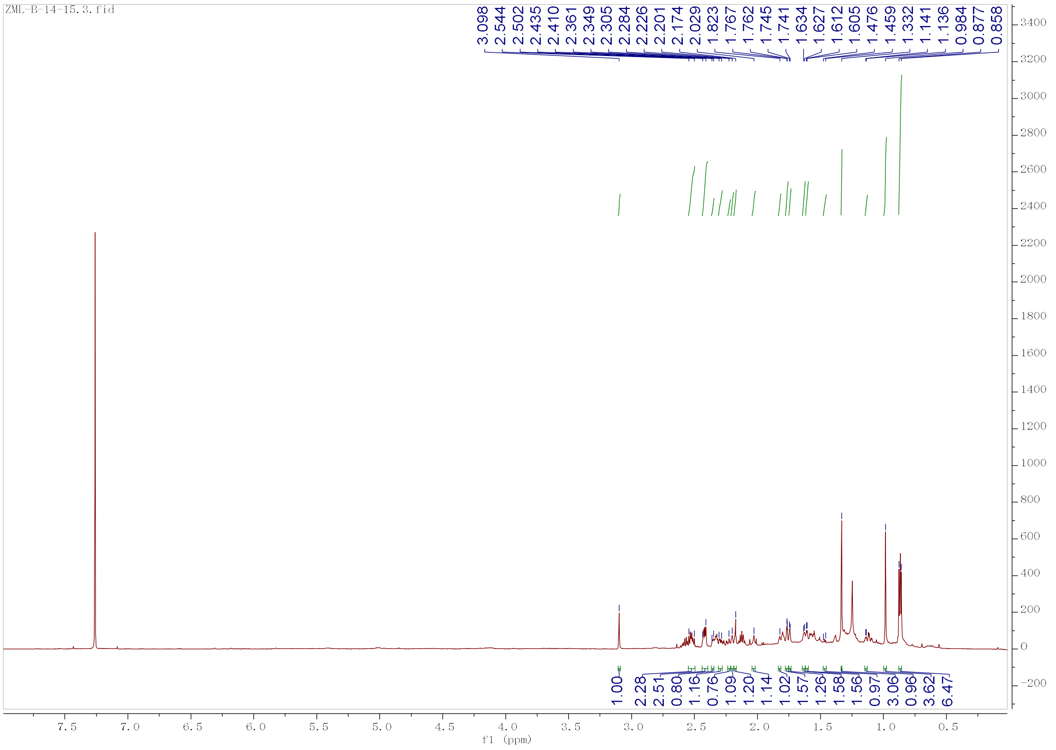

Figure S7.1 ^{1}H NMR spectrum of compound **7** (600 MHz, $CDCl_3$)

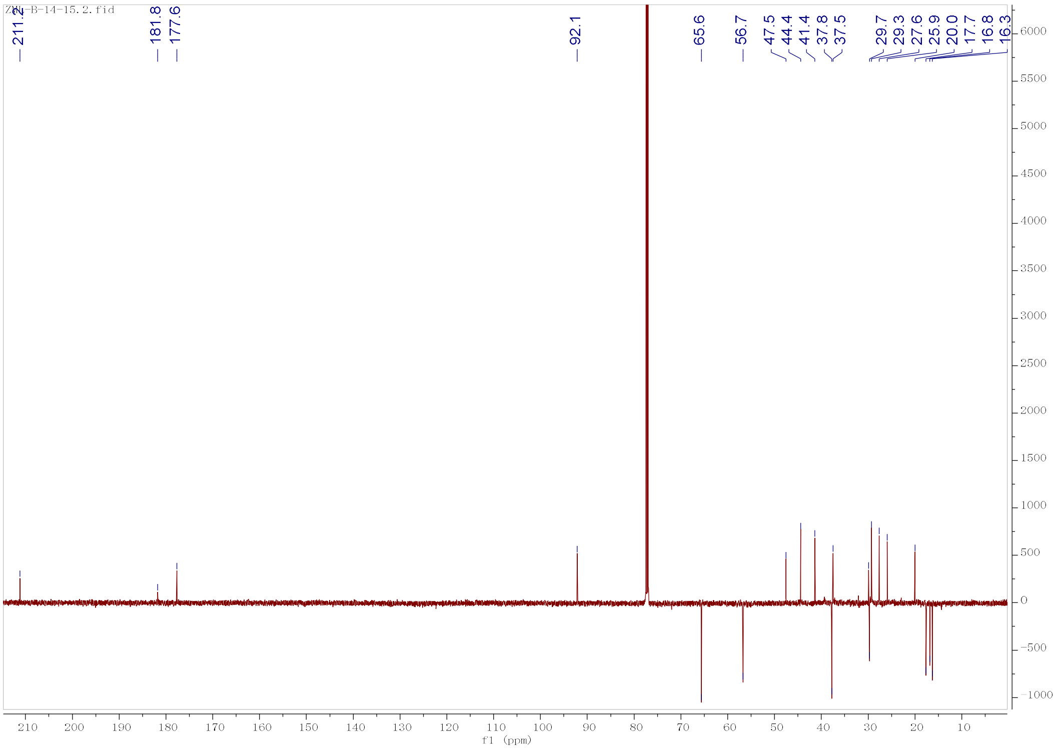

Figure S7.2 ^{13}C APT spectrum of compound **7** (150 MHz, $CDCl_3$)

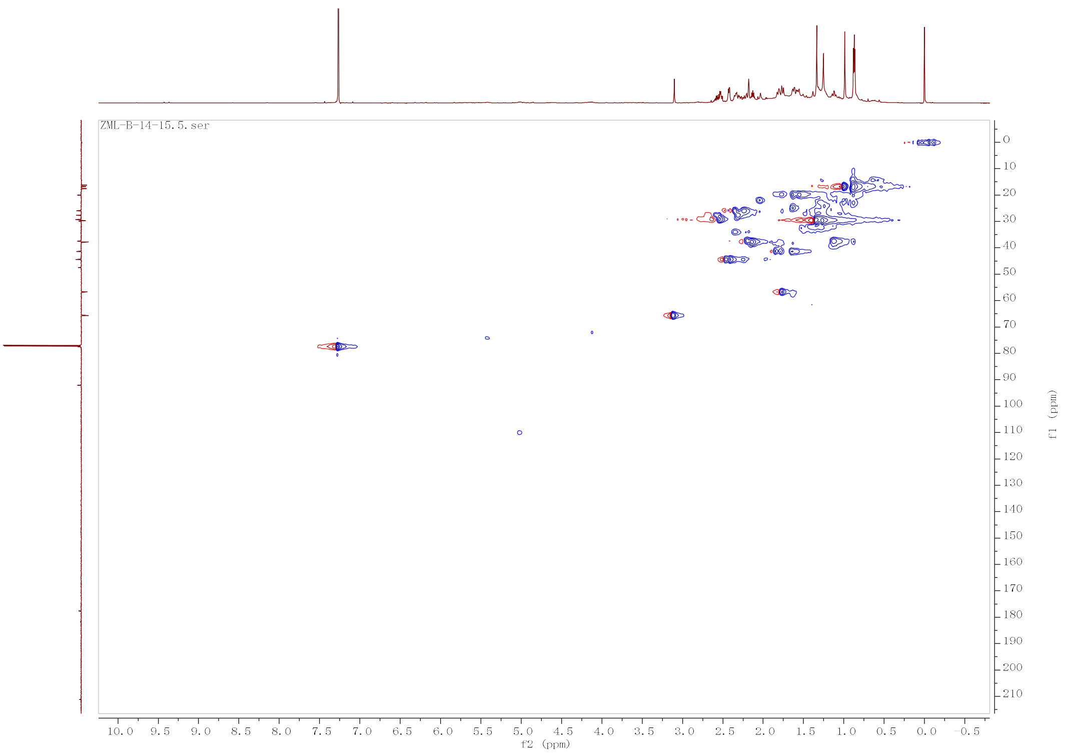

Figure S7.3 HSQC spectrum of compound **7**

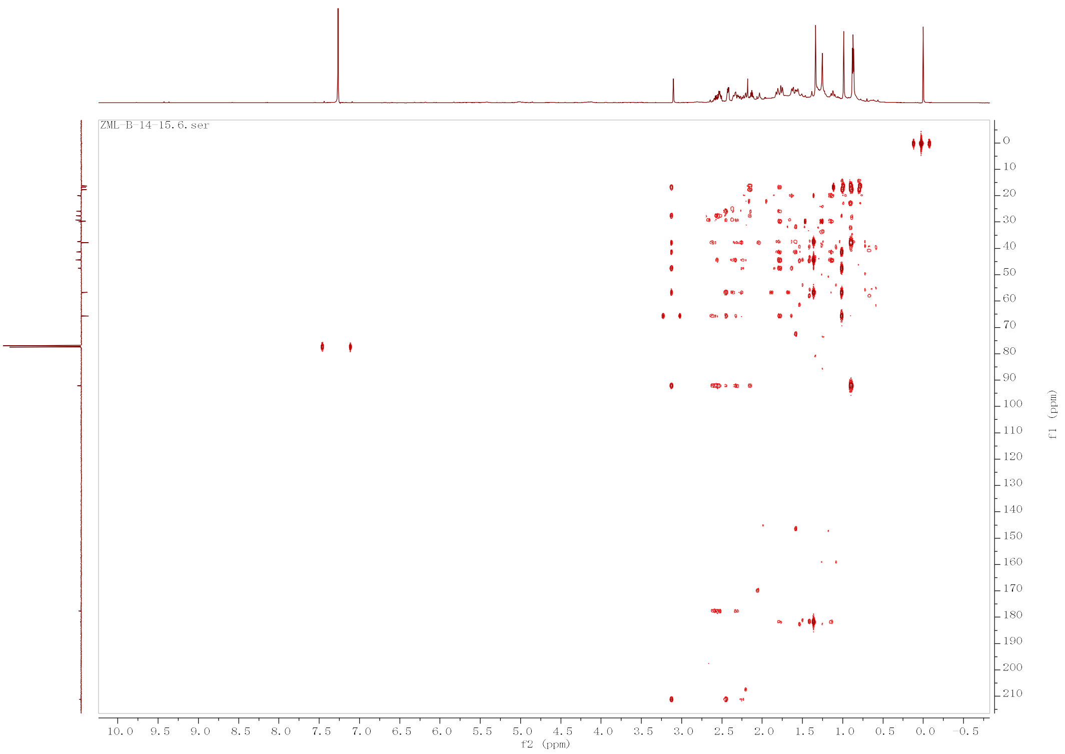

Figure S7.4 HMBC spectrum of compound **7**

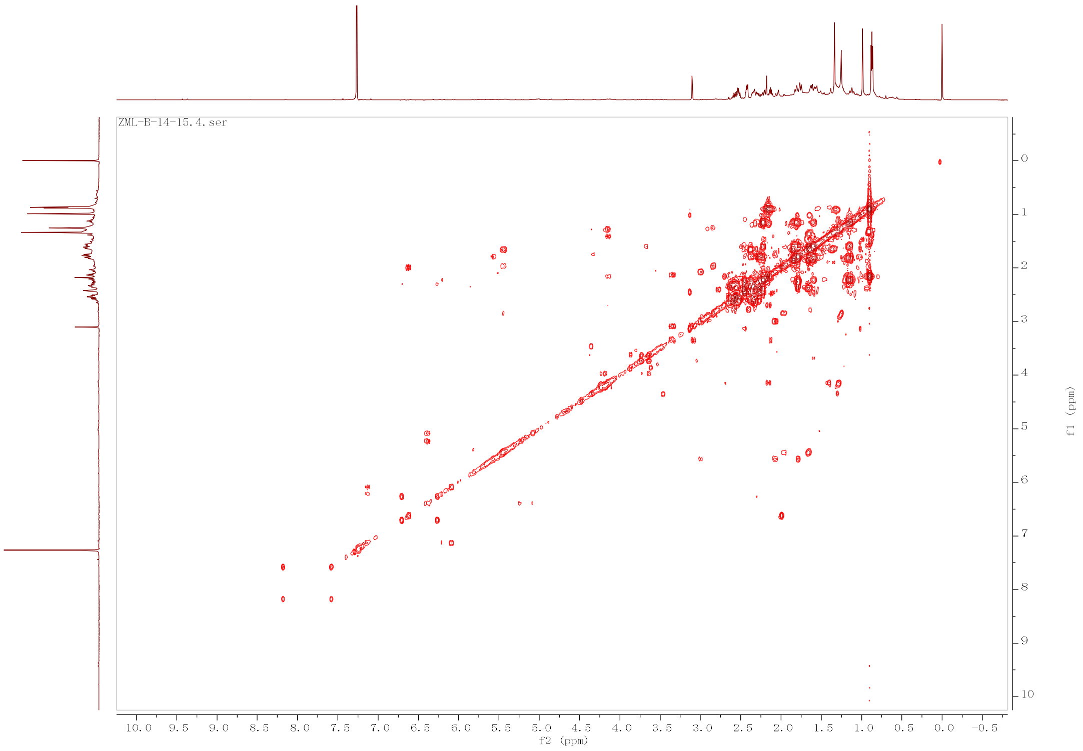

Figure S7.5 ^1H-^1H COSY spectrum of compound **7**

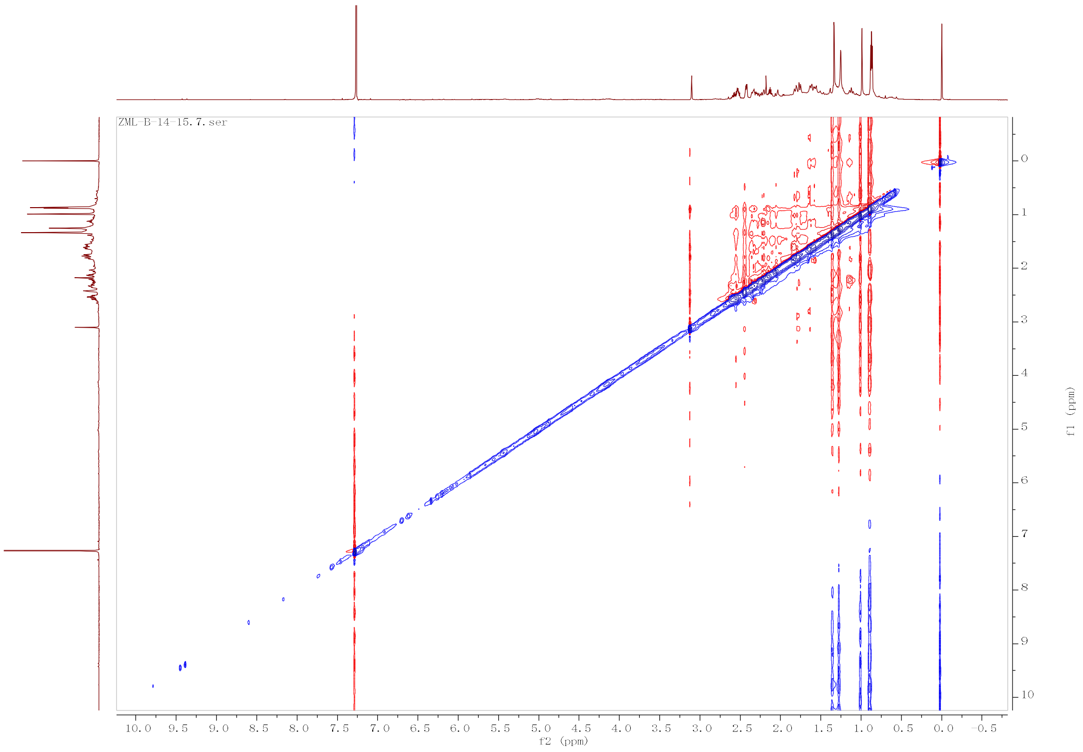

Figure S7.6 NOESY spectrum of compound **7**

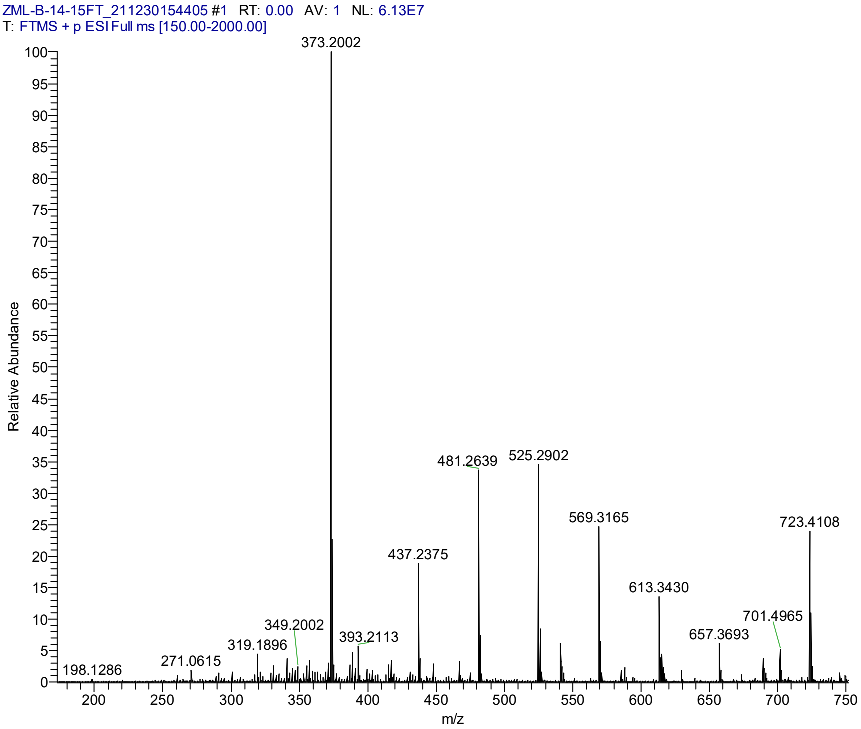

Figure S7.7 Positive ion HRESIMS of compound **7**

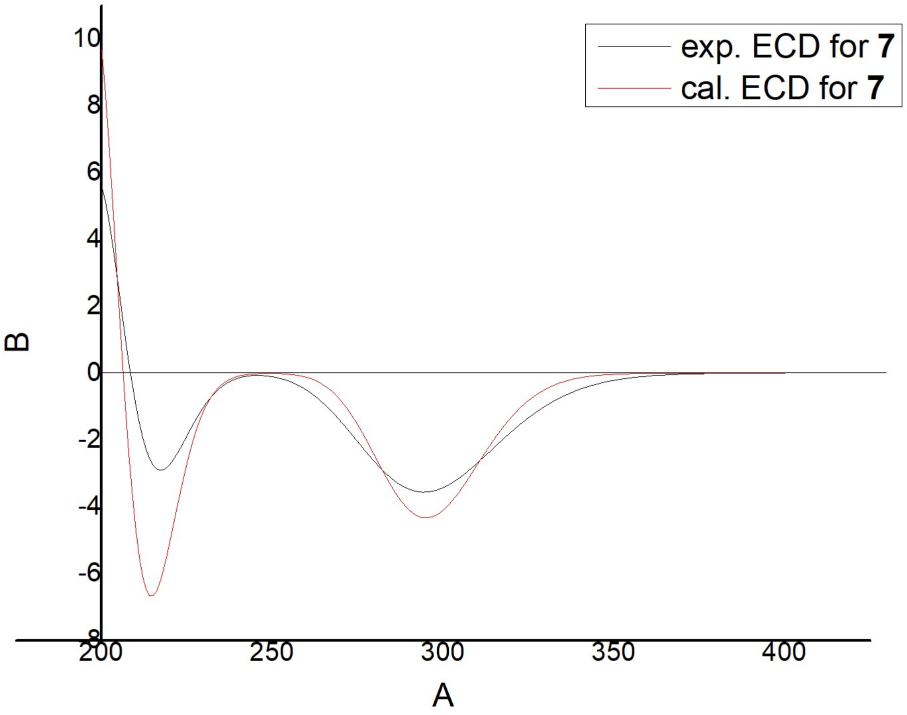

Figure S7.8 Experimental and calculated ECD spectra of compound **7**

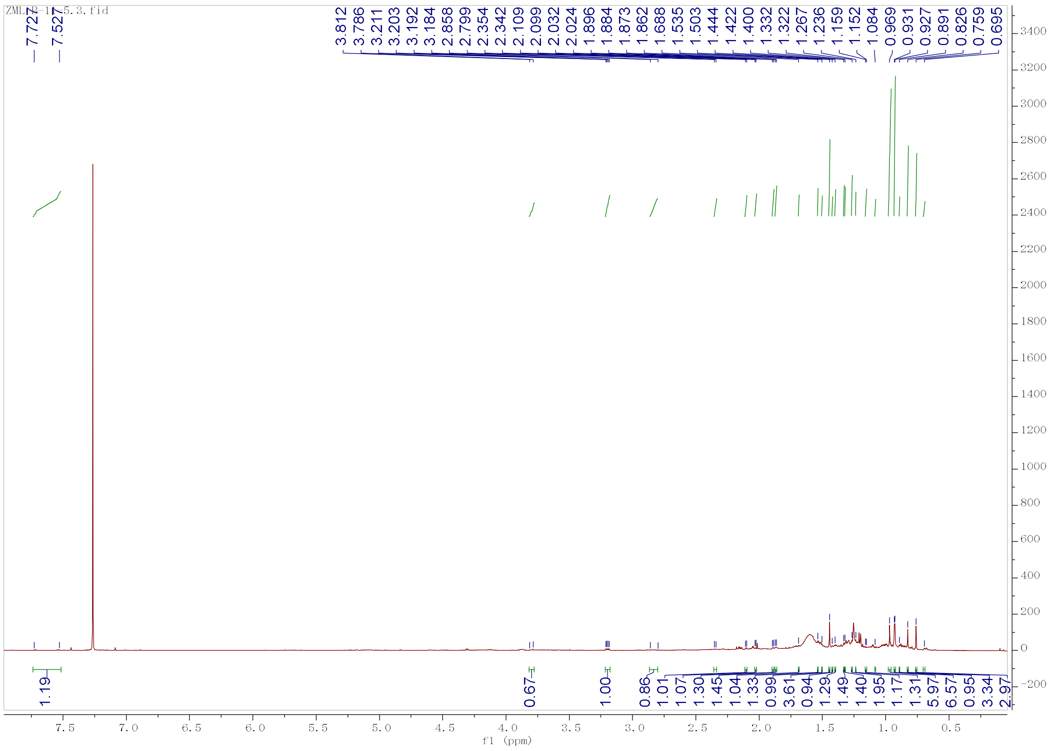

Figure S8.1 ^{1}H NMR spectrum of compound **8** (600 MHz, $CDCl_3$)

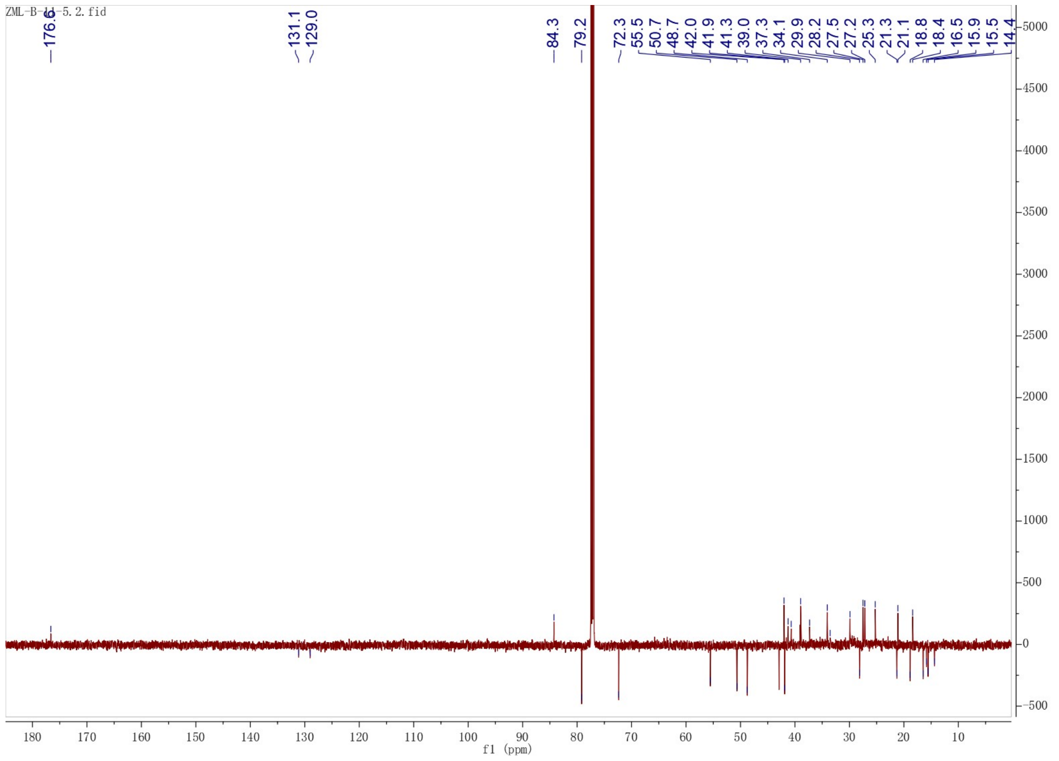

Figure S8.2 ^{13}C APT spectrum of compound **8** (150 MHz, $CDCl_3$)

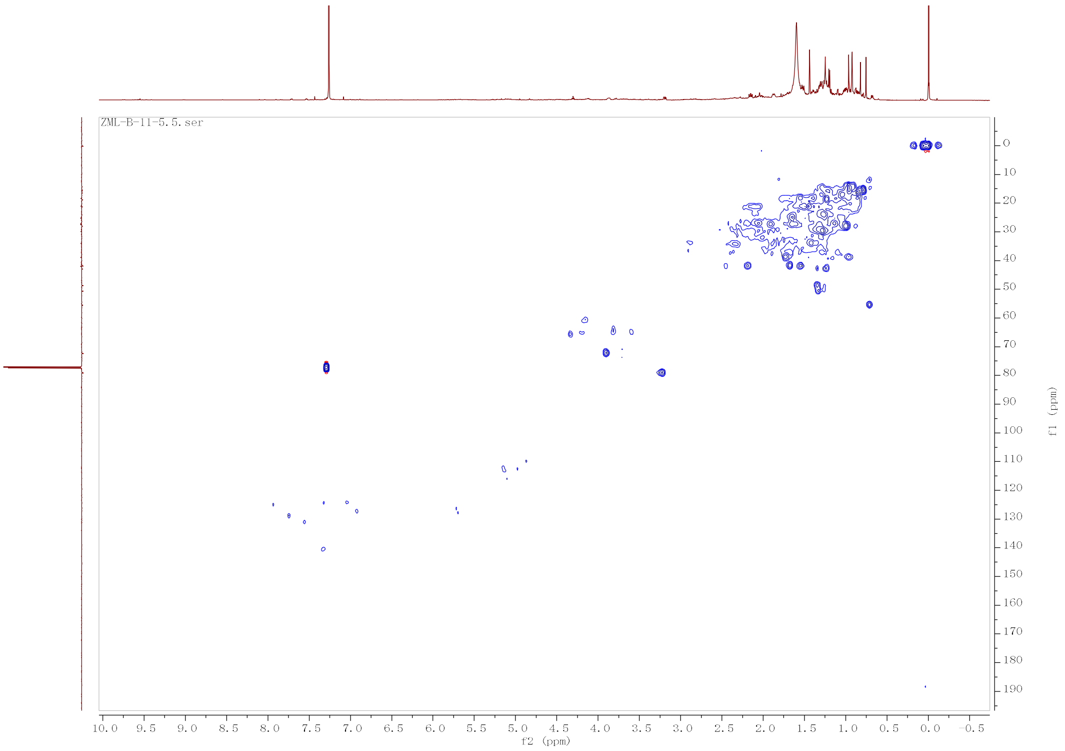

Figure S8.3 HSQC spectrum of compound **8**

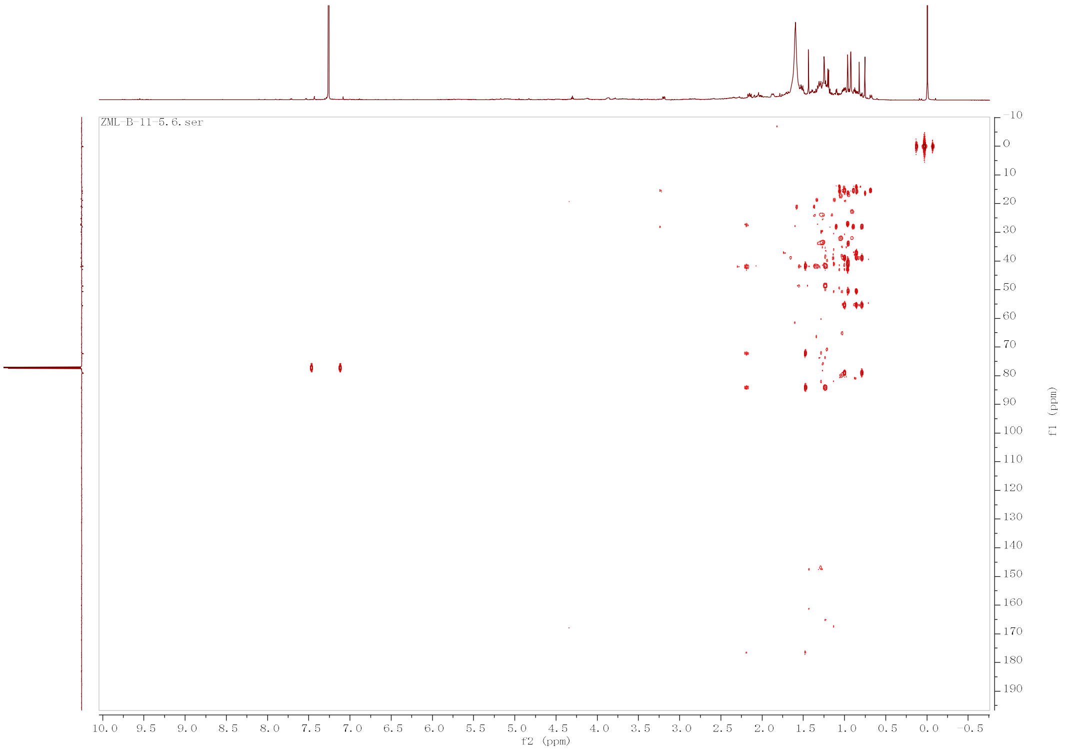

Figure S8.4 HMBC spectrum of compound **8**

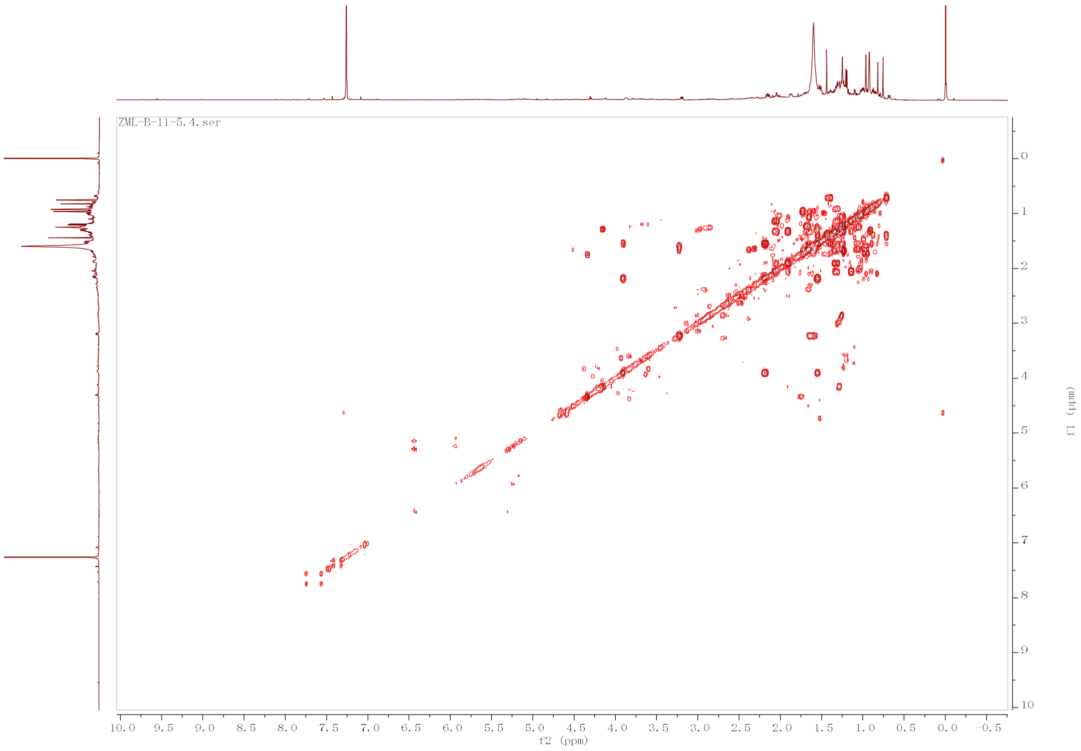

Figure S8.5 ^{1}H-^{1}H COSY spectrum of compound **8**

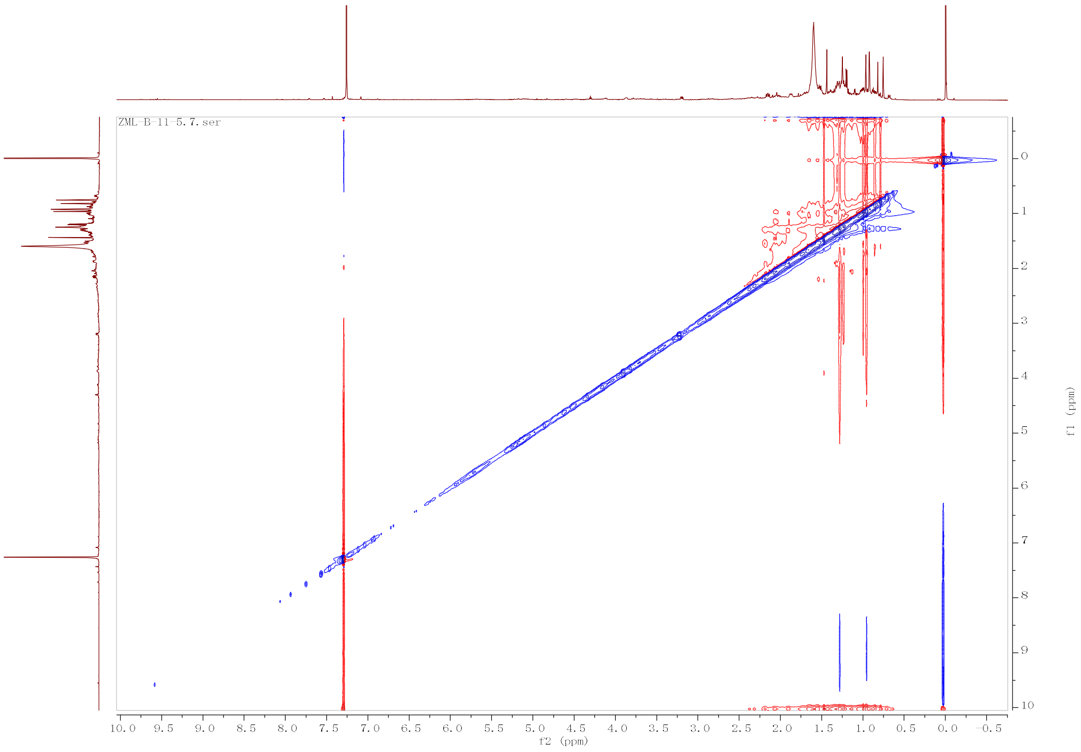

Figure S8.6 NOESY spectrum of compound **8**

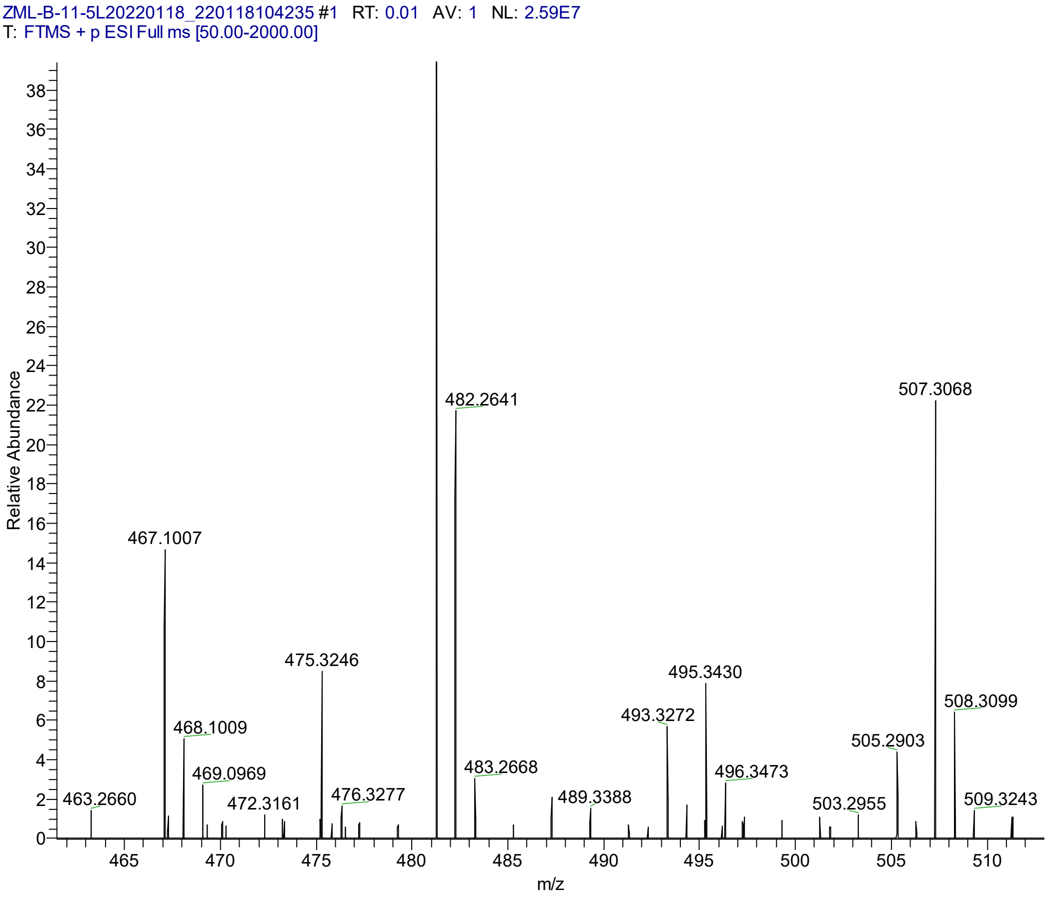

Figure S8.7 Positive ion HRESIMS of compound **8**
